# Hepatotoxicity assessment of innovative nutritional supplements based on olive-oil formulations enriched with natural antioxidants

**DOI:** 10.3389/fnut.2024.1388492

**Published:** 2024-05-15

**Authors:** Sofia I. Prodromou, Fani Chatzopoulou, Aikaterini Saiti, Alexandros Giannopoulos-Dimitriou, Loukia A. Koudoura, Anastasia A. Pantazaki, Dimitrios Chatzidimitriou, Vasilis Vasiliou, Ioannis S. Vizirianakis

**Affiliations:** ^1^Laboratory of Pharmacology, School of Pharmacy, Aristotle University of Thessaloniki, Thessaloniki, Greece; ^2^Laboratory of Microbiology, School of Medicine, Aristotle University of Thessaloniki, Thessaloniki, Greece; ^3^Labnet Laboratories, Department of Molecular Biology and Genetics, Thessaloniki, Greece; ^4^Laboratory of Biochemistry, Department of Chemistry, Aristotle University of Thessaloniki, Thessaloniki, Greece; ^5^Department of Environmental Health Sciences, Yale School of Public Health, Yale University, New Haven, CT, United States; ^6^Department of Health Sciences, School of Health and Life Sciences, University of Nicosia, Nicosia, Cyprus

**Keywords:** natural products, antioxidants, olive oil, vitamins, formulations, oxidative stress, cell cultures, HepG2

## Abstract

**Introduction:**

This study focuses on the assessment of extra virgin olive-oil and olive fruit-based formulations enriched with natural antioxidants as potential nutritional supplements for alleviating symptoms and long-term consequences of illnesses whose molecular pathophysiology is affected by oxidative stress and inflammation, such as Alzheimer’s disease (AD).

**Methods:**

Besides evaluating cell viability and proliferation capacity of human hepatocellular carcinoma HepG2 cells exposed to formulations in culture, hepatotoxicity was also considered as an additional safety measure using quantitative real-time PCR on RNA samples isolated from the cell cultures and applying approaches of targeted molecular analysis to uncover potential pathway effects through gene expression profiling. Furthermore, the formulations investigated in this work contrast the addition of natural extract with chemical forms and evaluate the antioxidant delivery mode on cell toxicity.

**Results:**

The results indicate minimal cellular toxicity and a significant beneficial impact on metabolic molecular pathways in HepG2 cell cultures, thus paving the way for innovative therapeutic strategies using olive-oil and antioxidants in dietary supplements to minimize the long-term effects of oxidative stress and inflammatory signals in individuals being suffered by disorders like AD.

**Discussion:**

Overall, the experimental design and the data obtained support the notion of applying innovative molecular methodologies and research techniques to evidently advance the delivery, as well as the scientific impact and validation of nutritional supplements and dietary products to improve public health and healthcare outcomes.

## 1 Introduction

Herbal mixtures and botanicals, historically exploited for their chemical constituents in nutritional and therapeutic interventions, are nowadays experiencing a resurgence in modern health contexts, aligning with the World Health Organization’s dietary recommendations (1). These natural sources offer essential nutrients and bioactive compounds such as vitamins, flavonoids, and minerals (2, 3). A diverse range of natural products, including vitamins, marine resources, and omega-3 fatty acids, shows promise in positively addressing symptoms of Alzheimer’s Disease (AD) by countering neuroinflammation and oxidative stress, key AD pathophysiological mechanisms (4–11). These compounds, much like phenolic compounds, tackle multiple pathways offering hope for delaying or preventing AD disease progression (12). In a landscape lacking effective pharmacological interventions for AD, dietary changes, supplementation, and organic products are gaining attention as potential alternatives.

Previous studies have highlighted the advantages of the Mediterranean diet, whereas recent work has shown that extra virgin olive oil (EVOO) and its constituents may affect health via interfering with cellular processes related to oxidative stress and inflammation (13–17). EVOO boasts a distinctive fatty acid profile dominated by monounsaturated fatty acids (MUFAs), particularly oleic acid (C18:1), which constitutes about 55–83% of its total fatty acids (18). EVOO is rich in tocopherols, with α-tocopherol being the predominant form. Tocopherols, a type of vitamin E, serve as potent antioxidants that protect against oxidative stress by scavenging free radicals and preventing lipid peroxidation (19). The synergistic action of MUFAs and tocopherols in EVOO contributes to its remarkable antioxidant capacity, which is associated with reduced oxidative stress and inflammation (20). Moreover, data has shown that oleic acid modulates inflammatory pathways by inhibiting the production of pro-inflammatory cytokines and enzymes, such as cyclooxygenase-2 (COX-2) and inducible nitric oxide synthase (iNOS) (21). Also, α-tocopherol exerts antioxidant and anti-inflammatory effects by neutralizing reactive oxygen species (ROS) and inhibiting the activation of inflammatory signaling pathways, such as nuclear factor-kappa B (NF-κB) (22). To this end, additional work has indicated that regular consumption of EVOO rich in oleic acid and tocopherols can reduce markers of oxidative stress and inflammation, thereby conferring protection against chronic diseases, including neurodegenerative disorders like AD (23–25).

The emergence of hepatotoxicity by nutritional supplements, especially of multi-ingredient content, represents a major healthcare and regulatory safety issue. To this end, the use of cell models and molecular techniques to assess hepatotoxicity via the application of commercially available gene expression arrays provides a valuable approach for the mechanistic evaluation of the safety of marketed nutritional supplements (26). Hepatocytes play a crucial role in metabolizing natural constituents, nutrients, and drugs in the organism, by transforming them into more water-soluble compounds for their elimination from the body. At the same time, however, this process in the liver can also lead to the formation of toxic metabolites (27, 28). Therefore, assessing hepatotoxicity in early stages is a vital aspect in the drug developmental process, as well as the marketed nutritional formulations. Hepatotoxicity analysis is essential to evaluate the potential harm that xenobiotics might cause to the liver and to ensure their safety before clinical use. It involves a comprehensive examination of liver function, including molecular and biochemical measurements, as well as histopathological changes pending the agent under investigation. Overall, by predicting and understanding hepatotoxicity is of paramount clinical and health significance since it minimizes the emergence of adverse effects from the liver, and thus it improves the safety and efficacy profiles of the developed products.

This work aimed to assess the hepatotoxicity of extra virgin olive oil and olive fruit-based formulations enriched with natural antioxidants. The utilization of HepG2 cells, a human hepatocellular carcinoma cell line, in this research serves the objectives of our study, since they represent a well-established model for assessing hepatotoxicity and investigating liver-specific metabolic molecular pathways (29, 30). In particular, the cellular viability and the proliferation potential were assessed in a concentration-and time-dependent manner in the model of human liver cancer HepG2 cells, whereas by applying targeted gene expression molecular analysis using real-time quantitative PCR of isolated RNA samples the hepatotoxicity safety was also evaluated at the molecular level. Importantly, by applying a commercially available hepatotoxicity assessment gene array, we evaluated the effect of the formulations tested in the expression of 84 genes covering multiple signaling pathways, like oxidative stress and of inflammatory processes. The bioinformatic analysis of the data obtained indicate that these multi-ingredient nutritional supplements exhibit a beneficial impact on the hepatic oxidative stress and inflammatory processes, a fact supporting the notion for additional research aiming to investigate any potential for alleviating symptoms in the pathogenesis of diseases, like AD and other neurodegenerative disorders.

## 2 Materials and methods

### 2.1 Cell cultures

The well-established human model of liver cancer HepG2 cells was used throughout this study. HepG2 cells were stored at-20°C and meticulously nurtured in a controlled environment (37°C; humidified atmosphere with 5% v/v CO2) using RPMI-1640 medium (Gibco™ RPMI 1640 Medium, Thermo Fisher Scientific), supplemented with 10% (v/v) Fetal Bovine Serum (FBS) (Gibco™ Fetal Bovine Serum, Thermo Fisher Scientific), along with 100 μg/mL of penicillin and 100 μg/mL of streptomycin (Gibco™ Pen-Strep, Thermo Fisher Scientific). To ensure the sustained logarithmic growth phase of the cell lines in culture, they were detached from the culture medium when approaching approximately 80% of confluence, which typically occurred every 2–3 days. This detachment was facilitated using trypsin–EDTA (0.25% w/v).

The synthesized formulations under scrutiny were previously prepared and solubilized/dissolved in either MilliQ H_2_O or Dimethyl Sulfoxide (DMSO) (Sigma Aldrich, Merck SA), and then stored at a controlled temperature of 4°C. In compliance with the experimental protocols, each cell culture was meticulously treated as delineated below. Notably, the concentration of DMSO in these treatments was strictly kept below or equal to 0.2% v/v.

### 2.2 Formulations under investigation

**SCHEME 1:**
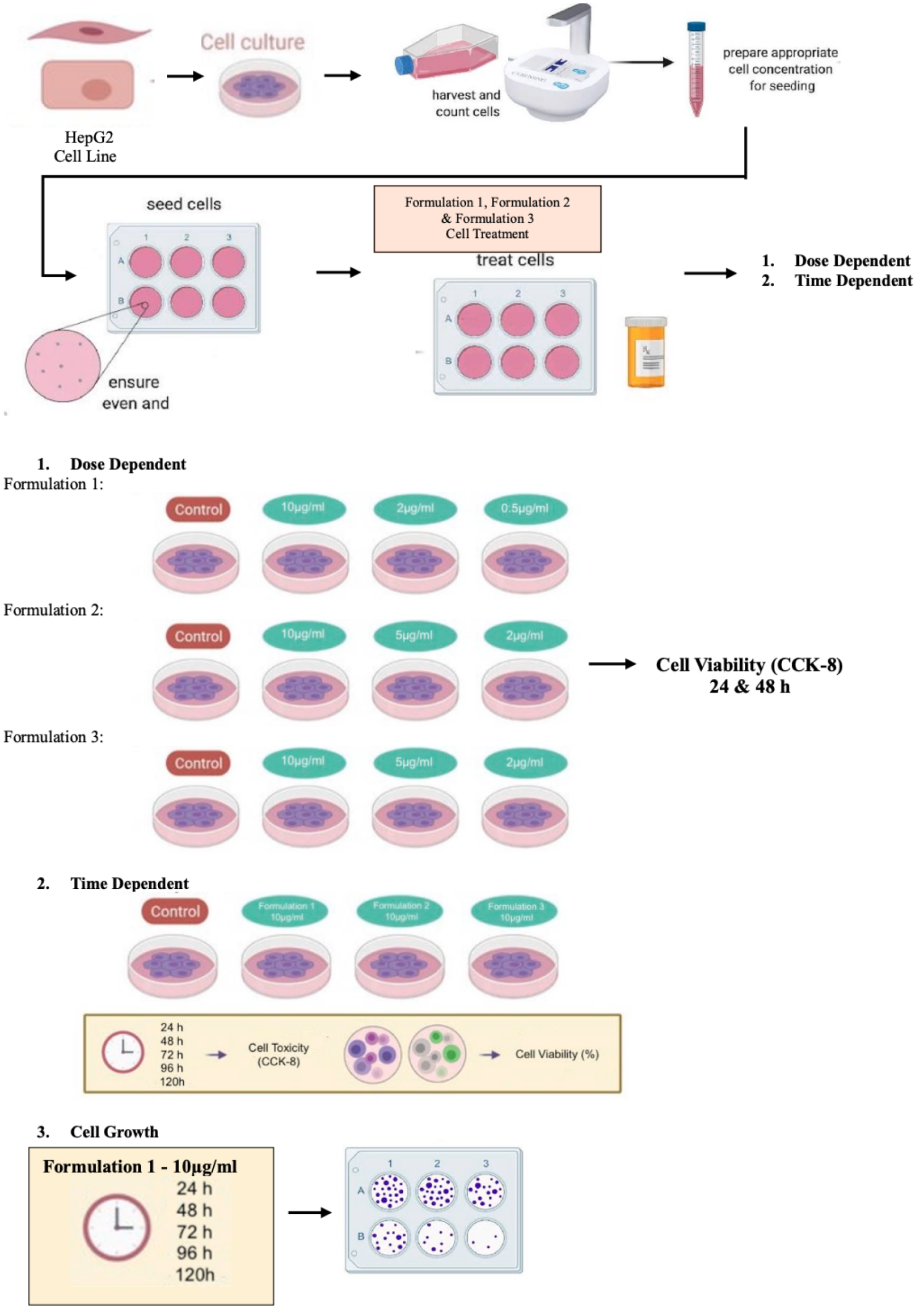
Diagrammatic depiction of the experimental procedure.

The experimental interventions, denoted as treatments, encompass two distinct formulations, each representing a unique product. The first formulation appears as a consumable liquid, comprising an extra virgin olive-oil foundation with a substantial phenolic content of a qualified market product from Yanni’s Olive Grove (Greece). This base is further enriched with assorted antioxidant components of qualified marketed ingredients (Vitamin A, E, C, oleuropein and DHA). The second and third treatment pertains to alternative formulations rooted in olive fruit (Yannis Olive Grove, Greece), wherein the antioxidant constituents (Vitamin E, oleuropein and DHA) are incorporated into either a cream or a chocolate paste utilized for stuffing the olive fruit. Regardless this first categorization, the three formulations under investigation are distinguished into two separate sub formulations (e.g., Formulation 1A & 1B etc.) according to their Active Pharmaceutical Ingredient (API) origin, with the letter A referring to natural extract API origin and the letter B to synthetic API origin. A diagrammatic depiction of the experimental process used to carry out the studies indicated in this work is shown in Scheme 1.

The pivotal antioxidant elements encompass a spectrum of compounds: vitamin A from a natural carrot extract (Avel, Greece), vitamin E from a natural mango extract (Avel, Greece), vitamin C from a natural orange extract (Avel, Greece), ellagitannins from a natural pomegranate extract (Avel, Greece), as well as oleuropein from a natural olive leaf extract (Avel, Greece), and the omega-3 fatty acid docosahexaenoic acid (DHA) from a natural sea algae extract (Polaris, France). All the APIs were prepared in stock solutions and from those it was taken the appropriate amount to prepare the final Formulations 1–3 in a manner that each separate API (e.g., Vitamin E) to be at a final concentration of 0.5 μg/mL, 2 μg/mL, and/or 10 μg/mL in which the HepG2 cell cultures were exposed. These scaled down concentrations were selected based on the recommended daily intake (DI) values of the APIs as prescribed by EMA guidelines (Table 1). As mentioned previously, to discern potential concentration-dependent toxicity, the Formulations underwent testing at varied concentrations of the constituent APIs in cell cultures, i.e., 0.5 μg/mL (four times lower concentration than DI), 2 μg/mL (DI) and 10 μg/mL (five times higher concentration than DI), and simultaneously, the product underwent a 24–120 h time-dependent evaluation to explore its potential cytotoxic profile over time. Moreover, as part of this evaluation of cellular effects of natural products, another formulation was included for comparison purposes. This counterpart (Formulation 1) consists of individual chemical forms of the active constituents, deviating from the natural fruit and vegetable extracts included in Formulations 2 and 3. Please note, to solve the weight problem of APIs in Formulations 2 and 3 the concentration scale was modified into 2 μg/mL (instead of 0.5 μg/mL used in Formulation 1), 5 μg/mL (instead of 2 μg/mL used in Formulation 1), and 10 μg/mL.

**Table 1:** Characteristics and ingredients of the formulations used in this study.

Name		Information of treatment	Ingredients	EMA DI of APIs (According to Greek regulation ΦΕΚ 3328-2017)	API’s Form
Formulation 1	Treatment 1A	Oral solution	Olive oil *(1,43 μL/mL)*	–	Natural extracts
			DHA *(3,5 mg/mL)*	–	
			Oleuropein *(3,5 mg/mL)*	–	
			Vitamin A *(70 mg/mL carrot extract)*	800 μg	
			Vitamin E *(7 mg/mL mango extract)*	12 mg	
			Vitamin C *(1,06 mg/mL orange extract)*	60 mg	
	Treatment 1B	Oral solution	Olive oil *(1,43 μL/mL)*	–	Pure APIs
			DHA *(3 mg/mL)*	–	
			Oleuropein *(3 mg/mL)*	–	
			Vitamin A *(0,6 mg/mL)*	800 μg	
			Vitamin E *(0,625 mg/mL) oil solution with 96% Vit E*	12 mg	
			Vitamin C *(0,6 mg/mL)*	60 mg	
Formulation 2	Treatment 2A	Olive fruit with flavor enhancer (banana cream)	DHA *(1,5 mg/mL)*	–	Natural extracts
			Vitamin E *(3 mg/mL mango extract)*	12 mg	
			Oleuropein *(1,5 mg/mL)*	–	
	Treatment 2B	Olive fruit with flavor enhancer (banana cream)	DHA *(1,5 mg/mL)*	–	Pure APIs
			Vitamin E *(0,3 mg/mL)*	12 mg	
			Oleuropein *(1,5 mg/mL)*	–	
Formulation 3	Treatment 3A	Olive fruit with flavor enhancer (chocolate cream)	DHA *(1,5 mg/mL)*	–	Natural extracts
			Vitamin E *(3 mg/mL mango extract)*	12 mg	
			Oleuropein *(1,5 mg/mL)*	–	
	Treatment 3B	Olive fruit with flavor enhancer (chocolate cream)	DHA *(1,5 mg/mL)*	–	Pure APIs
			Vitamin E *(0,3 mg/mL)*	12 mg	
			Oleuropein *(1,5 mg/mL)*	–	

DHA, docosahexaenoic acid; API, active pharmaceutical ingredients.

### 2.3 Cell propagation in established cell lines and cytotoxicity assessment

Cell Counting Kit-8 (CCK8, St. Louis, MO, Sigma-Aldrich) reagent was employed, as a regulatory approved method for testing cell viability (31). The HepG2 cell lines, once well-established, were introduced to a 96-well plate at an initial concentration of 8 × 10^3^ cells/mL and allowed to stabilize for 24 h before introducing the designated formulations (treatments). These formulations were scaled down to achieve the final concentration of (0.5, 2.0 and 10 μg API/mL) and subsequently incubated at 37°C in a 5% CO_2_ incubator for 24 h. To determine the half-maximal inhibitory concentration (IC_50_) of each treatment for every plate, the cells were allowed to proliferate for an additional 48 h, following which they were harvested through trypsinization. Post-harvest, the cell count (cell density; number of cells/mL) was ascertained with the aid of an optical microscope using Neubauer counting chambers.

To quantify cell toxicity, it was expressed as a percentage relative to that observed in the untreated control culture. Quantification of the formazan product, gauged by its absorbance at 450 nm, exhibited a direct correlation to the number of viable cells in the culture. Control wells were meticulously prepared under identical experimental conditions. Wells containing solely culture media without any treatment, 0.2% DMSO or MilliQ H_2_O were designated as controls.

The assessment of cell growth for treatments 1A and 1B was also undertaken by quantifying cell counts using the Neubauer counting chamber. HepG2 cells were seeded at a density of 8 × 10^3^ cells per well in 96-well culture plates, where they were maintained in RPMI-1640 media and allowed to adhere overnight. Following this, a span of 24 h elapsed before the cells were subjected to various concentrations of each treatment (0.5, 2.0 and 10 μg API/mL). For each concentration, treatments were applied in triplicate, thereby ensuring experimental rigor. Following treatment, cells were incubated at 37°C in a 5% CO_2_ incubator for 48 h. Upon completion of the treatment period, trypsinization was performed according to previously established protocols and assessment of cell growth was undertaken by quantifying cell counts using the Neubauer counting chamber and subsequently juxtaposing these counts with those from control wells. Control wells were meticulously established under equivalent experimental conditions.

### 2.4 RNA isolation and real-time quantitative polymerase chain reaction

Isolation of RNA from cell cultures subjected to the investigated formulations was performed using the manual procedure stipulated by the RNeasy Plus Micro Kit from Qiagen (Qiagen – Bio Analytica, Greece). This protocol was tailored for the extraction of cytoplasmic RNA. For subsequent analysis, a two-step qPCR approach was adopted, as described in the RT2 Profiler PCR Array human hepatotoxicity SYBR Green kit (Qiagen-SafeBlood BioAnalytica SA, Greece) manufacturer’s instructions. The utilization of acidic phenol facilitated the preferential retention of RNA in the upper aqueous phase, effectively curbing RNase activity. Post-extraction, the purity of the obtained RNA was ascertained using the Thermo Scientific NanoDrop 2000 spectrophotometer.

The cDNA synthesis reaction, vital for subsequent analyses, employed a template RNA of 200 ng and followed the established protocol of the QuantiTect Reverse Transcription Kit, (Qiagen-Bio Analytica, Greece). In pursuit of optimal amplification plots within the range of 35 to 40 Ct (Cycle threshold) for each gene under scrutiny, a 1:20 dilution was systematically carried out on samples.

### 2.5 Bioinformatic analysis of the differentially expressed genes data

The differentially expressed genes data were subjected to bioinformatic analysis aiming to clarify their biological activities and the molecular pathways in which they are involved. ClusterProfiler (v4.0.5) R package was utilized to perform the overrepresentation analysis for Gene ontology (GO) as per Biological Process (BP), Molecular Function (MF), and Cellular Component (CC). The pathway analysis based on the Reactome pathway database and Disease Ontology Semantic and Enrichment analysis (DOSE) were performed using ReactomePA (v1.36.0) and DOSE (v.18.3) packages in R.

### 2.6 Statistical analysis

The statistical analysis of the data was conducted using GraphPad Prism version 10.0.2(171), focusing on precise analysis techniques tailored to the experimental design. Specifically, two-way ANOVA tests were employed for dose-dependent evaluations, allowing for robust assessment of the effects of varying concentrations of the tested compounds. For time-dependent analyses, ordinary one-way ANOVA tests were utilized to discern any temporal trends. Graph design was also carried out using GraphPad Prism to effectively visualize the data.

## 3 Results

### 3.1 Cytotoxicity assessment

**Figure 1:**
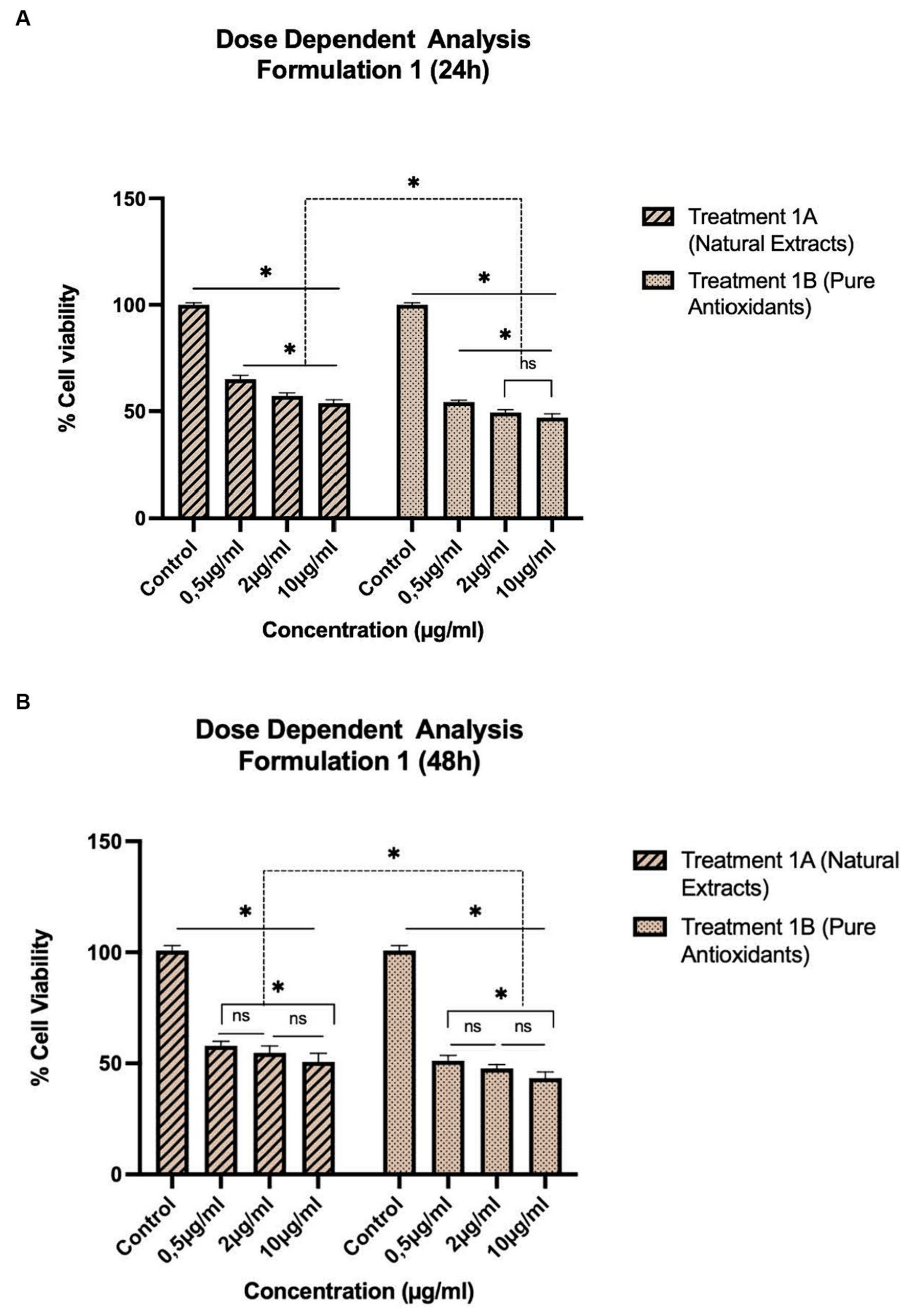
Concentration-dependent assessment of cellular viability of HepG2 cells exposed to Formulation 1 as measured with the CCK-8 assay. **(A)** Presents the data after 24 h-exposure, whereas **(B)** presents after 48 h-exposure. The data shown present statistical significance difference *p* < 0.05 compared to different treatments (*n* = 3) of two independent biological experiments.

**Figure 2:**
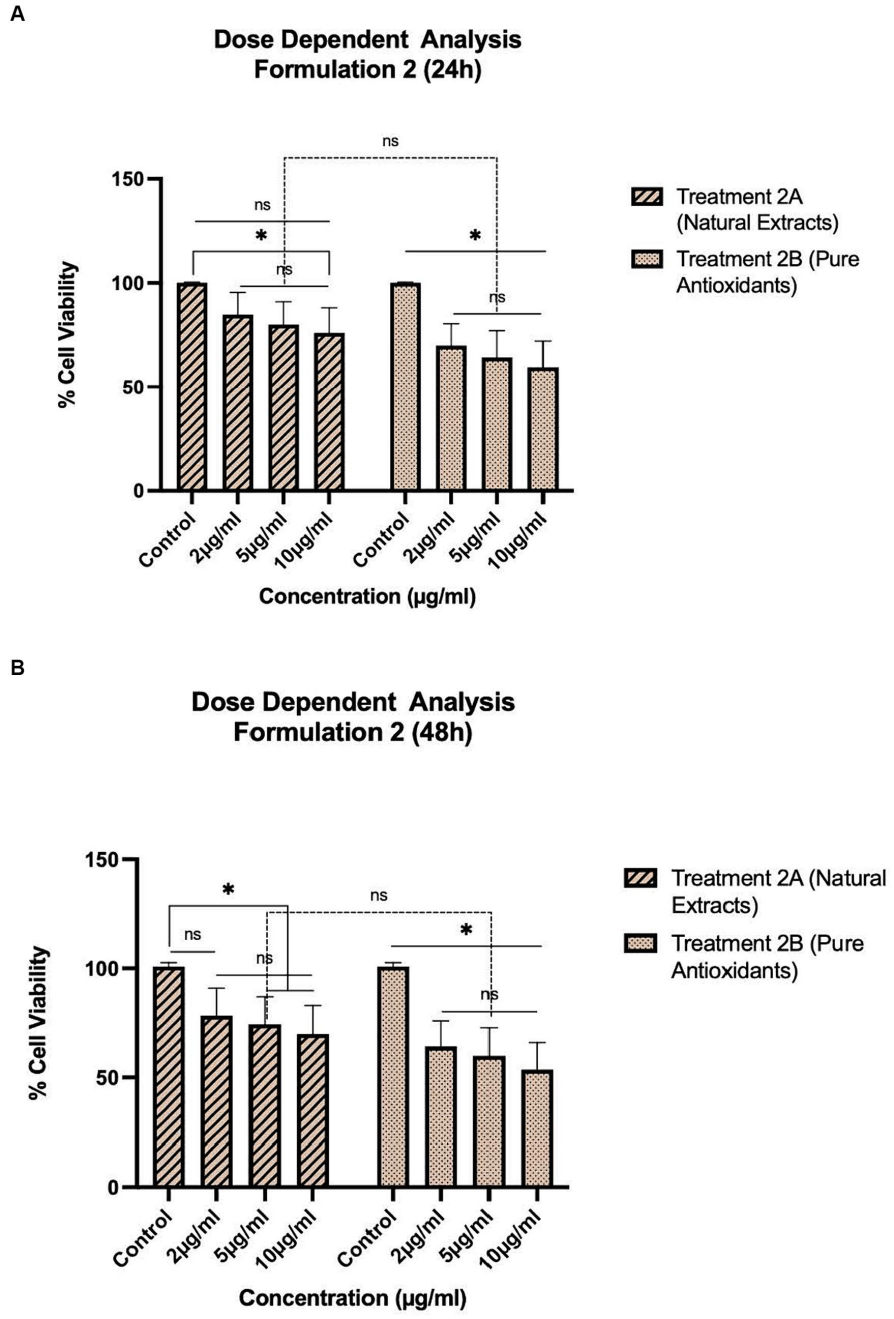
Concentration-dependent assessment of cellular viability of HepG2 cells exposed to Formulation 2 as measured with the CCK-8 assay. **(A)** Presents the data after 24 h-exposure, whereas **(B)** presents after 48 h-exposure. The data shown present statistical significance difference *p* < 0.05 compared to different treatments (*n* = 3) of two independent biological experiments.

**Figure 3:**
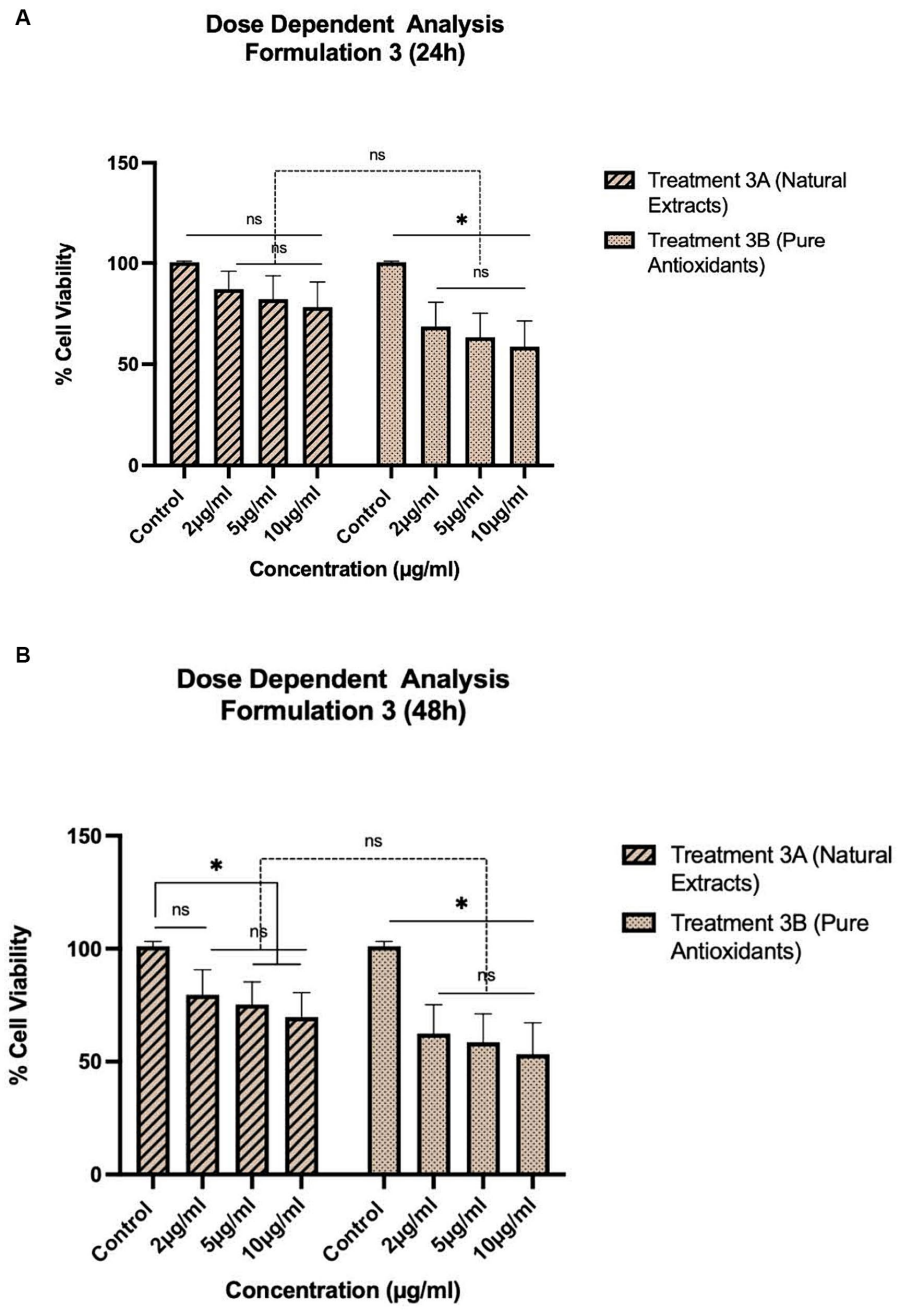
Concentration-dependent assessment of cellular viability of HepG2 cells exposed to Formulation 3 as measured with the CCK-8 assay. **(A)** Presents the data after 24 h-exposure, whereas **(B)** presents after 48 h-exposure. The data shown present statistical significance difference *p* < 0.05 compared to different treatments (*n* = 3) of two independent biological experiments.

**Figure 4:**
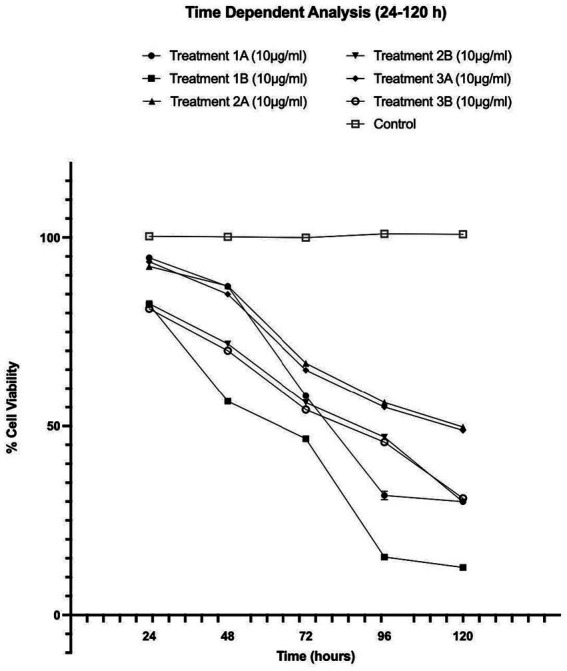
Time-dependent assessment (24–120 h) of cellular viability of HepG2 cells exposed to various treatments of Formulations 1, 2 and 3, as measured with the CCK-8 assay. The data present statistical significance difference *p* < 0.05 compared to different treatments (*n* ≥ 4) of two independent biological experiments.

The treatments, as described in Table 1, were selected for pharmacological evaluation along with pure olive oil and olive fruit extract used as control substances. To estimate the cell viability two separate experiments were conducted: one concentration dependent with selected concentrations and one time-dependent with 24–120 h evaluation. HepG2 cells were first incubated for 24 and 48 h with a concentration range of 0.5–10 μg/mL for treatments 1A, B (Figure 1) and a range of 2–10 mg/mL for treatments 2A, B (Figure 2) and 3A, B (Figure 3). After selecting the suitable concentration, the cells were incubated for 24–120 h with the treatments, proceeding to time a dependent assessment. In the time dependent experiment, the concentration of 10 μg/mL was selected to obtain more visible results (Figure 4).

As shown in Figures 1–4, the experimental results reveal several key findings regarding the impact of different formulations on cell viability, with the significance levels (*p* < 0.5) considered for the study play a crucial role in validating the observed trends and interpretations. Firstly, it is evident that formulations containing pure antioxidants (Formulations 1B, 2B & 3B) lead to a significant reduction in cell viability compared to those containing natural extract constituents (Formulations 1A, 2A & 3A). This disparity underscores the potency of pure antioxidants in inducing cytotoxic effects within the cellular environment, possibly due to their direct interaction with cellular components. Conversely, formulations with natural extract constituents exhibit comparatively higher cell viability, suggesting a milder cytotoxic profile attributable to their complex multi-nutrient composition and possibly beneficial synergistic effects of natural compounds present in the extracts.

Secondly, the analysis indicates a lack of substantial differences between the doses tested, highlighting a uniform response across the dosage spectrum. While this finding suggests a consistent cytotoxic effect irrespective of dosage, the decision to employ higher doses in subsequent experiments aims to elucidate more pronounced differences in cytotoxicity between formulations, thus providing a comprehensive understanding of their comparative efficacy.

Furthermore, the time-dependent assessment of cytotoxicity reveals notable trends between pure antioxidants and natural extracts. Specifically, formulations containing pure antioxidants exhibit more intense toxicity in viability over time compared to those with natural extracts. This observation is supported by the drastic drop in cell viability observed between 48 and 96 h of treatment with pure antioxidants, whereas natural extracts maintain a more stable reduction in cell viability over the same time-period.

**Figure 5:**
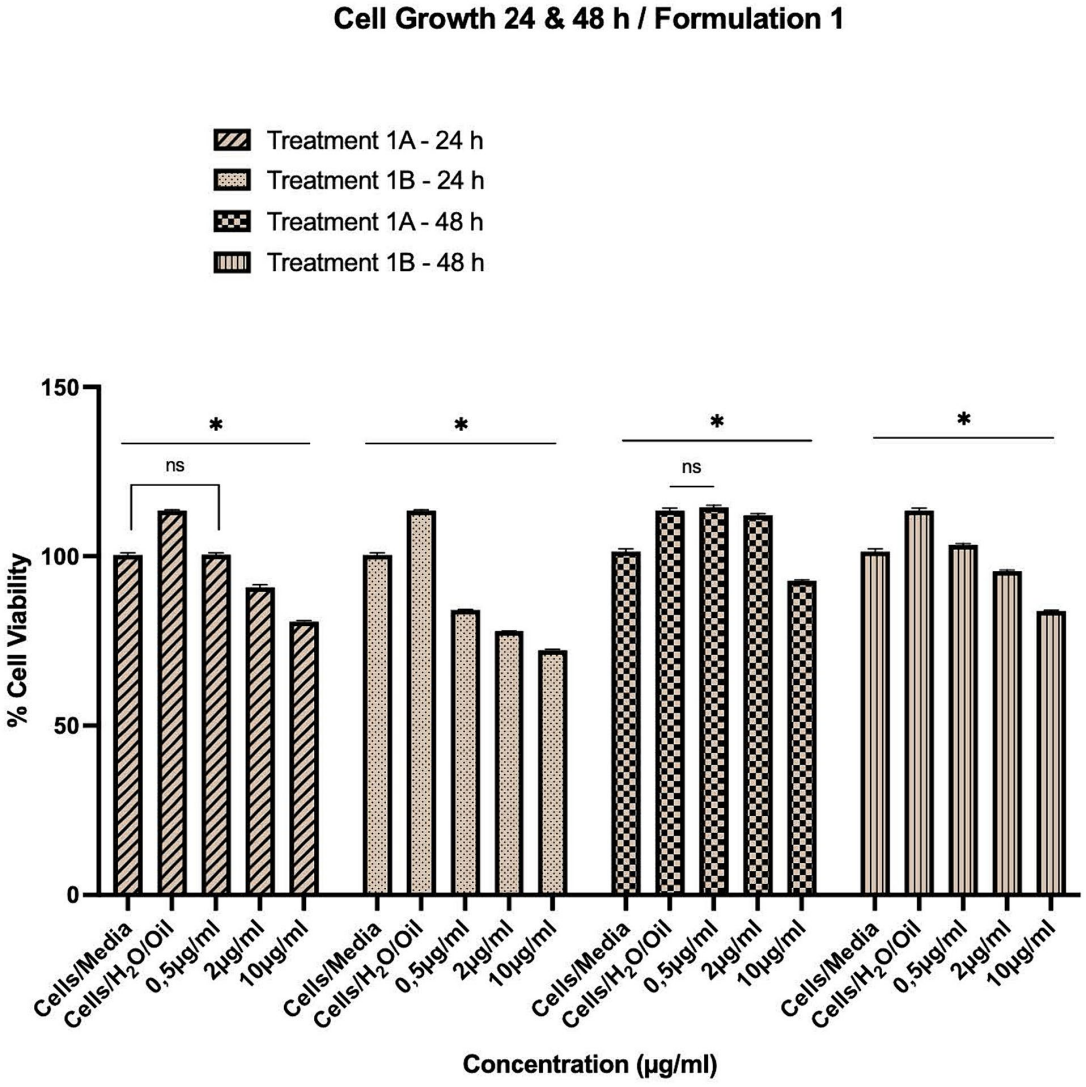
Concentration-and time-dependent assessment of HepG2 cell growth potential exposed to different treatments of Formulation 1, as measured with the Neubauer counting chambers. The data present statistical significance difference *p* < 0.05 compared to different treatments (*n* ≥ 4) of two independent biological experiments.

While cell viability provides information about the overall health and survival capacity of cells by measuring the activity of specific cellular enzymes, cell growth focuses on the proliferative potential to divide in culture, (cell-cycling capacity), by counting the actual number of cells accumulated over time. Both of parameters are crucial for understanding cellular responses to various treatments, including the assessment of cytotoxicity, xenobiotic efficacy, and cellular molecular physiology. In this case of the supplementary method of cytotoxicity assessment using the Neubauer counting chamber (Figure 5), while overall cell viability decreases over time across all formulations, the comparison with moderate decline in cell growth patterns suggests a moderate to minimal overall toxicity profile. Notably, regarding the cell growth assay which remains after 48 h-exposure above 85% in the highest doses of natural extract exposure and above 75% in the highest doses of pure antioxidants, indicating a relatively preserved cellular viability despite the observed effects in viability, with the statistically significance level (*p* < 0.5) to serve as a cornerstone in evaluating the reliability and credibility of these findings. This discrepancy between cell growth potential and cell viability capacity shows different cellular responses, a fact that underscores the complexity of the effects attributed to different formulations and highlights the need for comprehensive assessment to elucidate xenobiotic therapeutic potential and safety profiles.

The selection of appropriate control groups in the evaluation of hepatotoxicity is crucial for accurately assessing the effects of olive-oil-based formulations enriched with natural antioxidants. The inclusion of control groups consisting of untreated HepG2 cells and cultures of HepG2 cells treated with pure olive oil combined with water for injection serves as essential benchmarks for comparison. HepG2 cells without any treatment provide a baseline measure of cell viability and functionality, allowing for the evaluation of any changes induced by the tested formulations. Pure olive oil combined with water for injection, serving to uncover any solvent effects of the cells in all experiments, ensures that any observed effects can be attributed specifically to the active ingredients present in the formulations rather than the vehicle itself.

The rationale behind the selection of control substances and the criterion for their comparison is essential for strengthening the argument that natural extracts offer a more favorable toxicity profile compared to pure chemical counterparts. By including these control groups, the study ensures a reliable baseline for assessing the effects of the experimental formulations. This approach enables the evaluation of changes induced specifically by the active ingredients present in the formulations, independent of any potential effects from the vehicle. Additionally, comparative analysis with pure chemical compounds of vitamins and antioxidants allows for a direct comparison of the toxicity profiles between natural extracts and synthetic counterparts. This meticulous comparison aims to elucidate the potential benefits of natural antioxidants in mitigating toxicity, providing valuable insights into their safety and efficacy when used in multi-nutrient formulations.

### 3.2 Gene expression profiling assessment with the RT2 profiler PCR array using quantitative real-time polymerase chain reaction

Subsequently, in light of the aforementioned experiments, a reasoned deduction led to the incubation of HepG2 cells for molecular analysis of gene expression in the designated treatment groups (1A, 2A and 3A). This involved selecting the highest concentration of 10 μg/mL and incubating for 24 and 48 h to accentuate distinctions in gene expression following the isolation of total RNA. A comprehensive evaluation of 84 genes was undertaken to identify potential hepatotoxic biomarkers (Supplementary Table S4). Among them, 5 genes (ACTB, B2M, GAPDH, HPRT1 and RPLP0) served as housekeeping genes, in addition to positive, genomic DNA, and reverse transcription controls. The procedures for RNA extraction and quantitative real-time PCR were executed following the protocols prescribed by the Qiagen RNeasy and RT2 Profiler PCR Array human hepatotoxicity SYBR Green kit manufacturer.

The quantification of messenger RNA levels of target genes was carried out using the ΔΔCT methodology, with GAPDH and ACTB serving as reference genes for normalization purposes in subsequent analysis. Following normalization, data were represented as a fold change relative to the gene expression observed in the control group.

**Figure 6:**
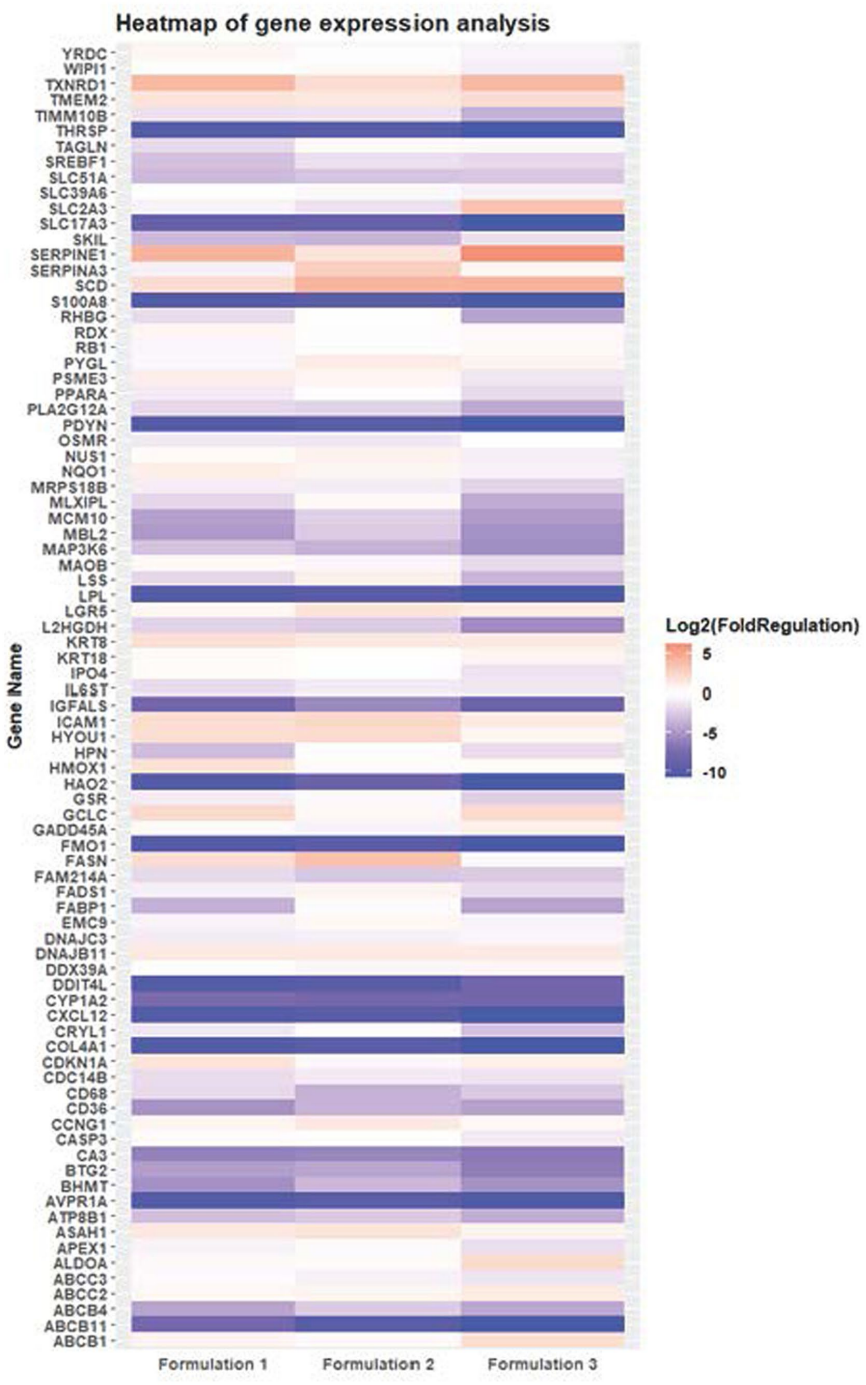
Heatmap representing the differentially expressed genes of HepG2 cells upon treatment with three distinct formulations (Formulation 1, 2 and 3) compared with control-untreated samples. The gene expression profiling assessment was conducted using the RT2 Profiler PCR Array Using Quantitative Real-time Polymerase Chain Reaction (RT-PCR). Rows represent gene names and columns display formulation index. Pink pixels: upregulated genes; Blue pixels: downregulated genes. The intensity of each color denotes the log2 fold-change values as obtained by the RT2 Profiler PCR Array Data analysis.

The findings of these results illuminate intricate alterations in the expression patterns of numerous genes within HepG2 cells upon treatment with the three distinct formulations (Figure 6). To comprehensively assess these alterations, the Ct values of the samples (Ctsample) were consistently found to be lower than those of the controls (Ctcontrol), indicative of a general enhancement in gene expression. This enhancement was quantified using the metric of Fold up-or Down-Regulation. According to statistical analysis, it was determined that Formulation 1 induced differential regulation in 60 genes of HepG2 cells, with 16 genes upregulated and 44 genes downregulated (Supplementary Table S1). Similarly, Formulation 2 led in differential regulation in 49 genes, with 15 genes upregulated and 34 genes downregulated (Supplementary Table S2). Formulation 3 exhibited differential regulation in 62 genes, with 13 genes upregulated and 49 genes downregulated (Supplementary Table S3). These findings were obtained by considering log2(Fold Change) values and setting the |log2(Fold Change)| threshold at 2, ensuring robust identification of differentially regulated genes.

Upon scrutinizing the specific genes influenced by each formulation, Formulation 1 displayed intriguing patterns of gene activity. Noteworthy genes such as ABCB11, AVPR1A, COL4A1, CXCL12, DDIT4L, FMO1, HAO2, IGFALS, LPL, PDYN, SLC17A3, THRSP, and S100A8 exhibited distinct regulatory patterns. These genes were linked to diverse metabolic pathways, including multidrug resistance, antidiuretic hormone receptor, collagen chain trimerization, apoptosis, cytochrome P450 enzymes, mTOR signaling, DNA damage repair, biotransformation, pharmacodynamics, lipid metabolism, insulin-like growth factor transportation, plasma lipoprotein assembly, cell cycle progression, sodium-dependent transport, and fatty acid metabolism. Notably, some genes, such as ABCB11, AVPR1A, and S100A8, displayed patterns of inactivity, while others, such as DDIT4L and SLC17A3, exhibited hyperactivity, underlining the intricate regulatory mechanisms at play.

In comparison, Formulations 2 and 3 demonstrated similar trends in gene regulation, although the magnitude of differential gene expression varied. Both formulations exhibited differential regulation in genes related to metabolic processes, including fatty acid and lipid metabolism and transportation, as well as pathways responsible for drug response and organic acid transportation. Notably, Formulation 3 exhibited more pronounced differential gene regulation, indicating a potentially greater impact on these pathways. Additionally, the analysis unveiled the overexpression of specific genes in all formulations. Genes such as DNAJB11, GCLC, ICAM1, KRT8, SCD, SEPRINA1, TMEM2, and TXNRD1 were consistently upregulated, suggesting their crucial roles in the cellular response to the formulations. Intriguingly, some genes exhibited overexpression patterns unique to specific formulations, further emphasizing the distinct effects of each formulation on gene expression.

Moreover, certain genes showed reduced expression levels in response to the formulations, affecting pathways such as phospholipid transportation, carbon metabolism, transcription, glycoprotein receptor activity, triglyceride metabolism, MST1 signaling, ATP synthesis, apoptosis kinase 2, eukaryotic genome replication, bile acid synthesis, acyl chain remodeling, and steroid metabolism. The intricate interplay of gene expression underpins the impact of these formulations on diverse metabolic pathways, with 27 pathways affected by Formulation 1 and 35 pathways affected by Formulations 2 and 3.

In conclusion, this comprehensive analysis provides a detailed understanding of how these formulations modulate gene expression patterns in HepG2 cells. While Formulation 1 demonstrated a notable impact on metabolic pathways, apoptosis, and various cellular processes, as evidenced by the differential regulation of numerous genes, Formulations 2 and 3 exhibited similar trends, albeit with differences in the magnitude of gene expression changes. Notably, these formulations affected pathways associated with fatty acid metabolism, transportation, and drug responses. The findings underscore the potential relevance of these formulations in the regulation of crucial metabolic pathways and cellular functions, holding promise for applications in addressing metabolic disorders and related conditions. Further investigations are warranted to explore the underlying molecular mechanisms and therapeutic implications of these formulations.

**Figure 7:**
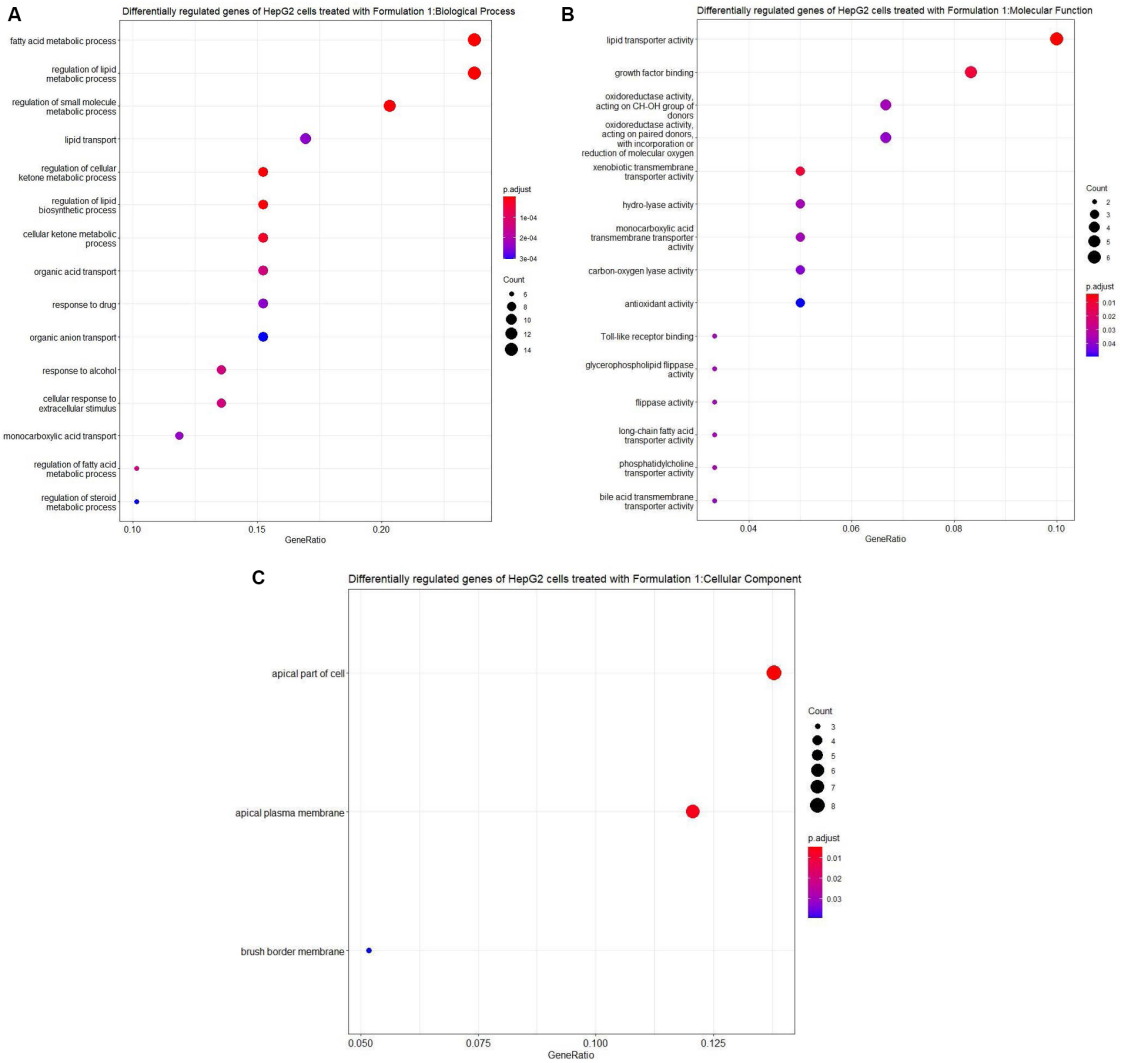
Gene ontology (GO) enrichment analysis of the differentially regulated genes of HepG2 cells treated with Formulation 1. **(A)** Top-15 significantly enriched gene ontology (GO) biological process (BP) terms associated with the differentially regulated genes of HepG2 cells treated with Formulation 1. The gene ratio and statistical significance (*p*-value <0.05, following Benjamini and Hochberg’s adjustment method) are also depicted. **(B)** Top-15 significantly enriched gene ontology (GO) molecular function (MF) terms associated with the differentially regulated genes of HepG2 cells treated with formulation 1. The gene ratio and statistical significance (*p*-value <0.05, following Benjamini and Hochberg’s adjustment method) are also depicted. **(C)** Top-3 significantly enriched gene ontology (GO) cellular component (CC) terms associated with the differentially regulated genes of HepG2 cells treated with Formulation 1. The gene ratio and statistical significance (*p*-value <0.05, following Benjamini and Hochberg’s adjustment method) are also depicted.

**Figure 8:**
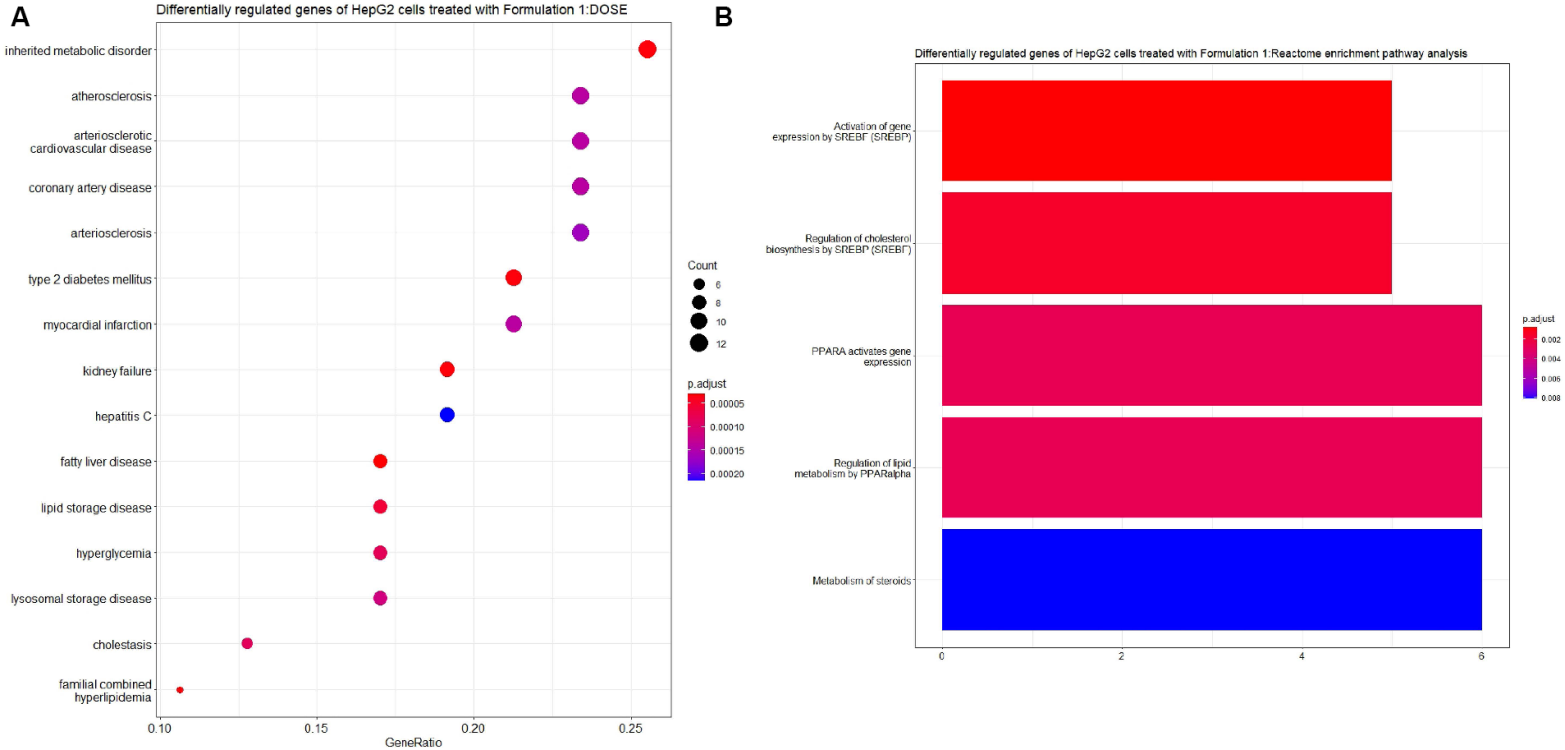
Disease ontology (DOSE) and pathway enrichment analysis of the differentially regulated genes of the cells treated with Formulation 1. **(A)** Top-15 significantly enriched disease ontology (DOSE) terms associated with the differentially regulated genes of HepG2 cells treated with Formulation 1. The gene ratio and statistical significance (*p*-value <0.05, following Benjamini and Hochberg’s adjustment method) are also depicted. **(B)** The REACTOME pathway enrichment analysis on the differentially regulated genes of HepG2 cells treated with formulation 1. The top-5 statistically significant pathways are listed, and their colors correspond to the adjusted *p*-values.

The examination of the HepG2 cell gene expression profiles following treatment with Formulation 1 yielded insightful results. For Formulation 1 (Figures 7, 8), the analysis revealed that differential gene regulation significantly impacted a range of biological processes. Notably, these processes encompass fatty acid metabolism, metabolic regulation, and lipid transportation. This suggests that Formulation 1 may exert its influence on these critical cellular activities, potentially affecting lipid homeostasis and metabolic regulation. In terms of molecular functions, the study found that lipid transporter activity, growth factor binding, and oxidoreductase activities exhibited significant modulation. These molecular functions are essential for various cellular processes, including the transportation and metabolism of lipids, as well as growth factor-mediated signaling. In contrast, functions like Toll-like receptor binding and glycerophospholipid activity were influenced to a lesser extent, implying that Formulation 1 may have a more substantial impact on certain aspects of cellular function. The top cellular component terms associated with the differentially regulated genes were related to the apical part of the cell and the plasma membrane, indicating that these cellular structures may be particularly affected by Formulation 1. Among the fifteen significantly enriched disease ontology (DOSE) terms associated with differentially regulated genes, prominent observations include a substantial association with metabolic disorders, exemplifying the formulation’s impact on crucial cellular metabolic pathways. Additionally, cardiovascular and artery diseases exhibited noteworthy enrichment, raising implications for potential cardiovascular effects. The presence of type-2 diabetes, kidney failure, and myocardial infarction in the list underscores the multifaceted consequences of Formulation 1 on cellular processes. Furthermore, while less pronounced, the formulation displayed effects on diseases such as fatty liver, lipid storage disease, cholestasis, and hyperlipidemia, indicative of its potential to influence lipid-related disorders. In terms of REACTOME pathway enrichment analysis, the top five statistically significant pathways align with the observed gene expression changes. These pathways, closely associated with SREBF gene expression, encompass regulation of cholesterol biosynthesis, regulation of lipid metabolism, and PPARA gene expression, collectively supporting Formulation’s 1 impact on lipid-related molecular processes.

**Figure 9:**
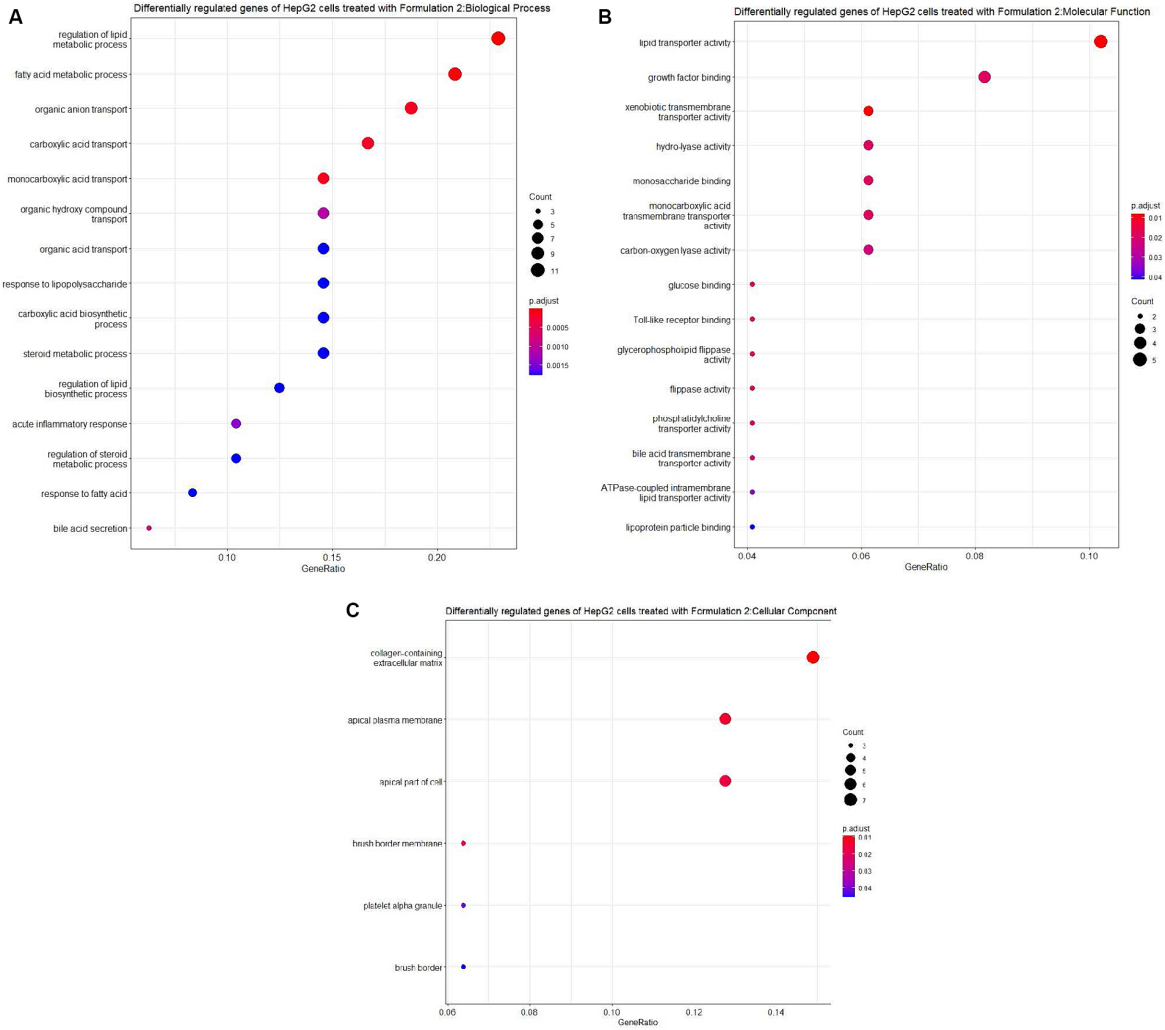
Gene ontology (GO) enrichment analysis of the differentially regulated genes of HepG2 cells treated with Formulation 2. **(A)** Top-15 significantly enriched gene ontology (GO) biological process (BP) terms associated with the differentially regulated genes of HepG2 cells treated with Formulation 2. The gene ratio and statistical significance (*p*-value <0.05, following Benjamini and Hochberg’s adjustment method) are also depicted. **(B)** Top-15 significantly enriched gene ontology (GO) molecular function (MF) terms associated with the differentially regulated genes of HepG2 cells treated with Formulation 2. The gene ratio and statistical significance (*p*-value <0.05, following Benjamini and Hochberg’s adjustment method) are also depicted. **(C)** Top-6 significantly enriched gene ontology (GO) cellular component (CC) terms associated with the differentially regulated genes of HepG2 cells treated with Formulation 2. The gene ratio and statistical significance (*p*-value <0.05, following Benjamini and Hochberg’s adjustment method) are also depicted.

**Figure 10:**
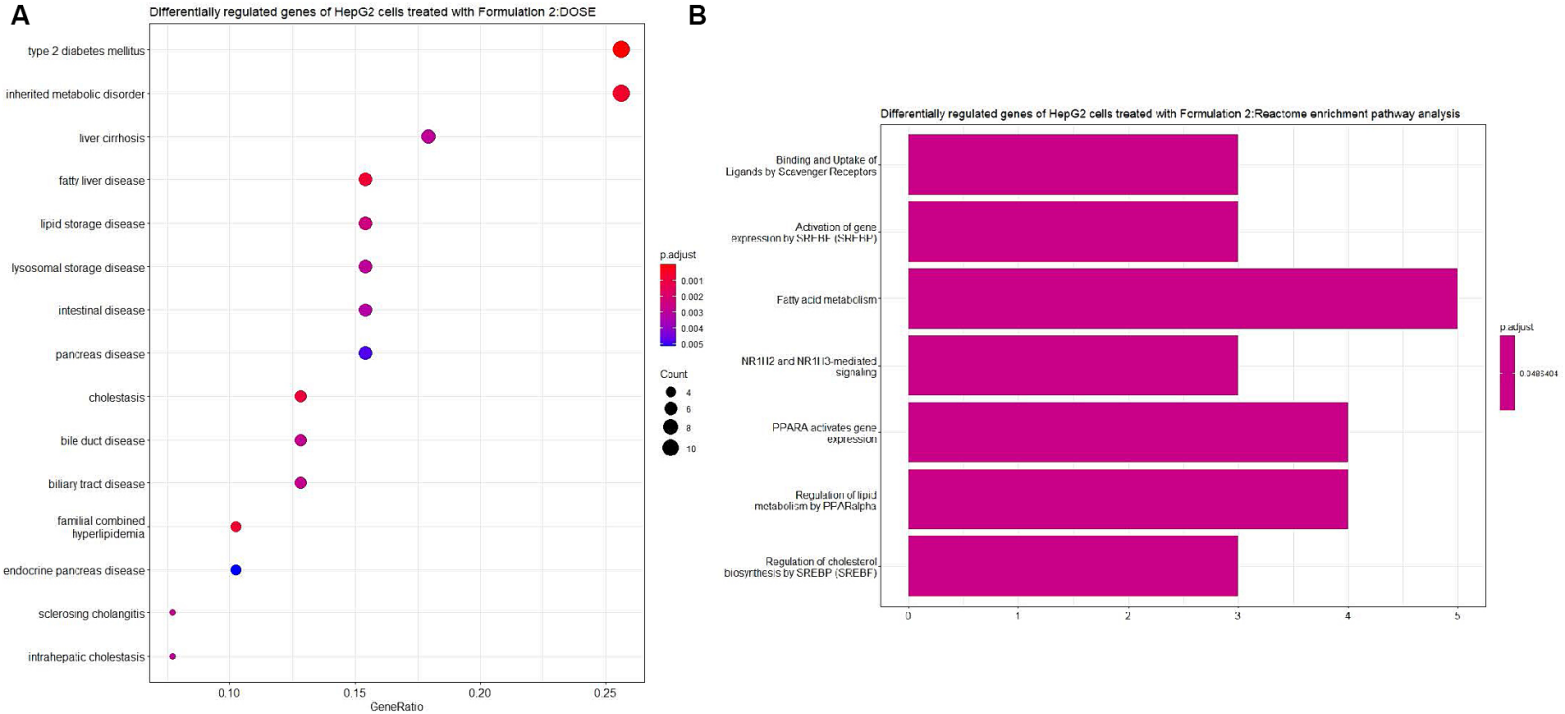
Disease ontology (DOSE) and pathway enrichment analysis of the differentially regulated genes of HepG2 cells treated with Formulation 2. **(A)** Top-15 significantly enriched disease ontology (DOSE) terms associated with the differentially regulated genes of HepG2 cells treated with Formulation 2. The gene ratio and statistical significance (*p*-value <0.05, following Benjamini and Hochberg’s adjustment method) are also depicted. **(B)** The REACTOME pathway enrichment analysis on the differentially regulated genes of HepG2 cells treated with Formulation 2. The top-7 statistically significant pathways are listed, and their colors correspond to the adjusted *p*-values.

Moving on to Formulation 2 (Figures 9, 10), similar trends in biological processes were observed, with a focus on fatty acid and lipid metabolism and transportation. However, in terms of molecular functions, Formulation 2 appeared to affect a different spectrum of activities. Notably, it had more genes affecting transmembrane activities, which are crucial for cellular transport processes. Conversely, molecular functions like glucose binding were less affected, likely influenced by the cellular components associated with the collagen-containing extracellular matrix and the apical part of the cell and plasma membrane. These findings suggest that Formulation 2 may have a distinct influence on cellular transport processes and extracellular matrix interactions. In terms of disease ontology, Formulation 2 showed strong associations with type 2 diabetes, metabolic disorders, liver and intestinal disorders, and lipid storage diseases. These disease associations provide insights into the potential therapeutic applications of Formulation 2 in addressing these health conditions. In the Reactome pathway analysis, pathways linked to ligand and scavenger receptors, fatty acid, and lipid metabolism, and SREBP and PPARA expression and biosynthesis were highlighted. These pathways shed light on the potential mechanisms through which Formulation 2 may exert its effects on cellular processes.

**Figure 11:**
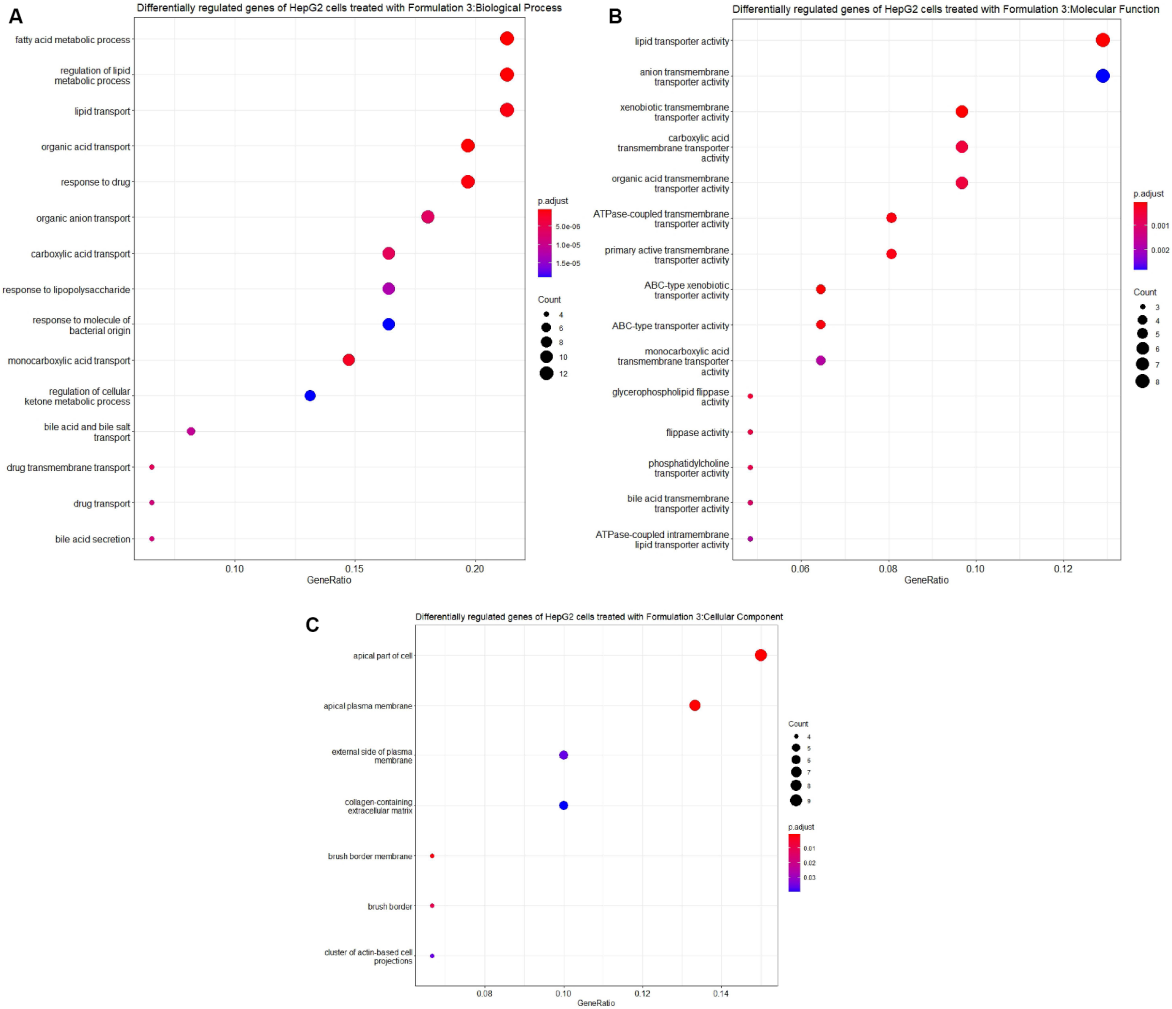
Gene ontology (GO) enrichment analysis of the differentially regulated genes of HepG2 cells treated with Formulation 3. **(A)** Top-15 significantly enriched gene ontology (GO) biological process (BP) terms associated with the differentially regulated genes of HepG2 cells treated with Formulation 3. The gene ratio and statistical significance (*p*-value <0.05, following Benjamini and Hochberg’s adjustment method) are also depicted. **(B)** Top-15 significantly enriched gene ontology (GO) molecular function (MF) terms associated with the differentially regulated genes of HepG2 cells treated with Formulation 3. The gene ratio and statistical significance (*p*-value <0.05, following Benjamini and Hochberg’s adjustment method) are also depicted. **(C)** Top-7 significantly enriched gene ontology (GO) cellular component (CC) terms associated with the differentially regulated genes of HepG2 cells treated with Formulation 3. The gene ratio and statistical significance (*p*-value <0.05, following Benjamini and Hochberg’s adjustment method) are also depicted.

**Figure 12:**
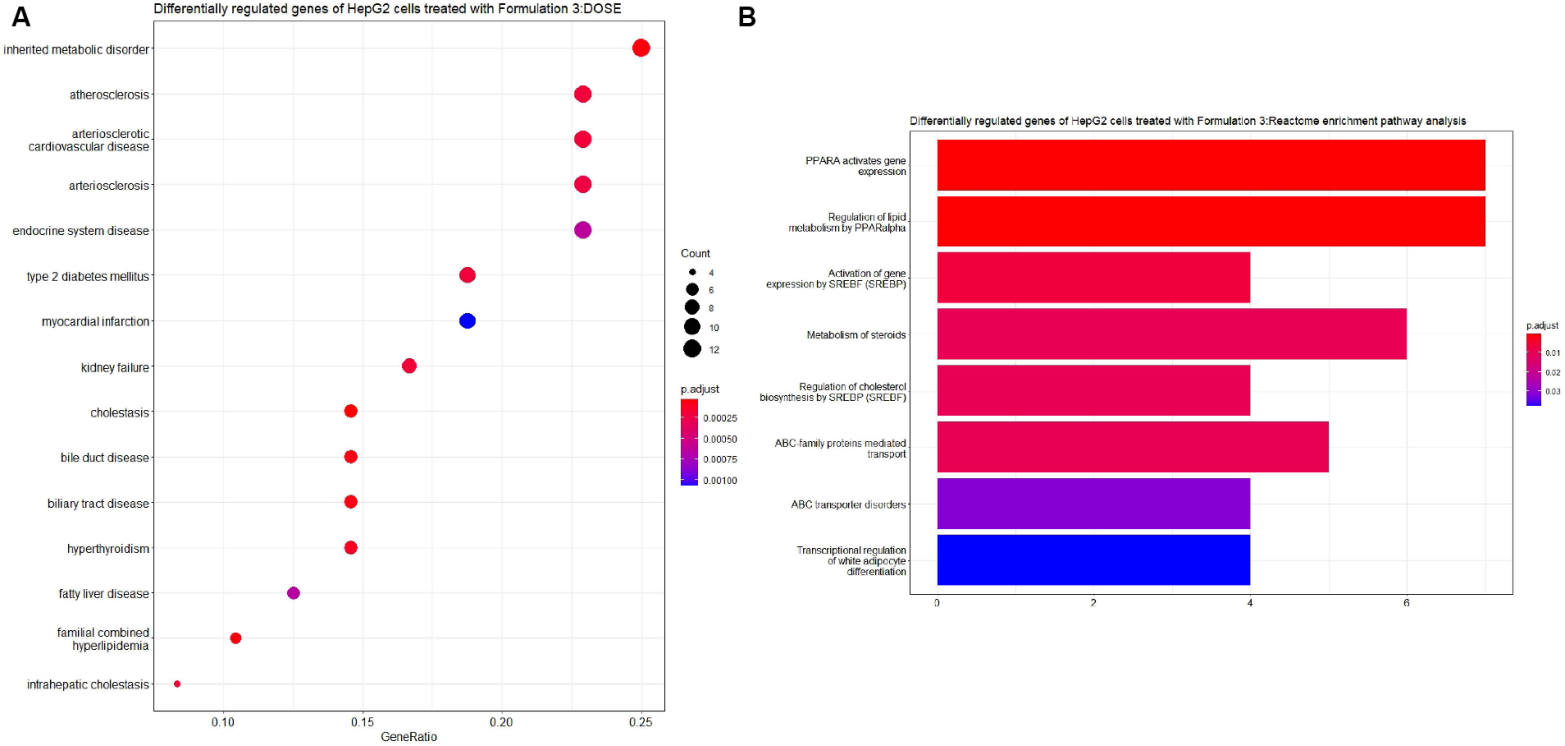
Disease ontology (DOSE) and pathway enrichment analysis of the differentially regulated genes of HepG2 cells treated with Formulation 3. **(A)** Top-15 significantly enriched disease ontology (DOSE) terms associated with the differentially regulated genes of HepG2 cells treated with Formulation 3. The gene ratio and statistical significance (*p*-value <0.05, following Benjamini and Hochberg’s adjustment method) are also depicted. **(B)** The REACTOME pathway enrichment analysis on the differentially regulated genes of HepG2 cells treated with Formulation 3. Top-8 statistically significant pathways are listed, and their colors correspond to the adjusted *p*-values.

Lastly, upon the treatment of HepG2 cells with Formulation 3 (Figures 11, 12), it appeared that a larger population of genes affected the biological processes related to fatty acid and lipid metabolism and transportation. Additionally, pathways responsible for the response to drugs and organic acid transportation were influenced. This suggests that Formulation 3 may have a substantial impact on cellular processes related to lipid metabolism and transportation, which are critical for maintaining cellular homeostasis. In terms of molecular functions, lipid, organic acid, and anion transportation activity showed significant influence, followed by ATPase-coupled ABC-type transportation through cellular membranes. These molecular functions are essential for cellular transport processes and ion balance. Lastly, functions like phosphatidylcholine and lipid membrane transportation seemed to be affected to a lesser extent. The top cellular component terms included the apical part of the cell, the plasma membrane, and the collagen-containing extracellular matrix. These cellular components are central to cellular structure and function. In terms of disease ontology, Formulation 3 presented similarities with Formulation 1, with top 15 significantly enriched disease ontology terms. These findings imply that Formulation 3 may share commonalities with Formulation 1 in terms of potential therapeutic applications. In the Reactome pathway analysis, lipid metabolism and PPARA gene expression were listed as the top-enriched pathways, followed by the metabolism of steroids, ABC family protein transportation, and SREBP cholesterol biosynthesis. These pathways provide insights into the potential mechanisms through which Formulation 3 may influence cellular processes.

In summary, this comprehensive analysis elucidated the differential effects of three distinct formulations on the gene regulation of HepG2 cells. While commonalities emerged in terms of biological processes impacted, each formulation exhibited unique effects on molecular functions and cellular components, highlighting the importance of formulation-specific considerations. Formulation 1 appeared to influence lipid metabolism, regulation of small molecules, and lipid transportation, with notable effects on lipid transporter activity and growth factor binding. Formulation 2, on the other hand, exhibited a distinctive influence on transmembrane activities and cellular matrix interactions, with associations with type 2 diabetes and metabolic disorders. Lastly, Formulation 3 demonstrated a substantial impact on fatty acid and lipid metabolism, as well as transportation processes, alongside potential implications for drug responses. These findings underscore the need for tailored approaches when assessing the potential therapeutic applications of these formulations. Future research and clinical investigations are warranted to harness their unique properties, potentially advancing treatments for various disorders.

## 4 Discussion

This study’s primary focus was cell toxicity evaluation in hepatic cell lines, with the results revealing a concentration-dependent reduction in cell viability over time, suggesting a moderate cytotoxicity associated with the 2 day-exposure (48 h) in culture. Intriguingly, formulations containing antioxidant components in their natural extract form demonstrate lower toxicity compared to their chemically pure counterparts, emphasizing the proficiency of natural extracts in minimizing toxic effects. Time-dependent analysis reveals dynamic fluctuations in cell viability, with an initial decline followed by stability in toxicological activity. In the context of the supplementary cytotoxicity evaluation method utilizing the Neubauer counting chamber (Figure 8), although overall cell viability declines over time across all formulations, the comparison with the moderate reduction in cell growth patterns suggests a moderate to minimal overall toxicity profile. Notably, in the cell growth assay, cell viability remains above 85% in the highest doses of natural extract exposure and above 75% in the highest doses of pure antioxidants, indicating relatively preserved cellular viability despite the observed cytotoxic effects. This discrepancy between cytotoxicity and cell viability underscores the intricate nature of cellular responses to various formulations and underscores the importance of comprehensive assessments to elucidate their therapeutic efficacy and safety profiles.

In the landscape of new drug development, the imperative of gene analysis has become increasingly pivotal in shaping pre-trial assessments. Gene analysis allows researchers to scrutinize how a drug interacts with specific genes, providing insights into potential variations in efficacy and adverse reactions among different patient populations. Moving to the results of the aforesaid-mentioned gene analysis, the formulations exhibit a broad influence on gene expression patterns in HepG2 cells. While all formulations promote the expression of examined hepatotoxicity genes, they also modulate specific genes related to inflammation, tissue homeostasis, immune surveillance, tumor growth, and metabolic processes. Delving deeper into the results of gene analysis in this study, Formulation 1 elicited distinct regulatory effects on 60 genes within HepG2 cells, resulting in the upregulation of 16 genes and the downregulation of 44 genes. Likewise, Formulation 2 prompted differential regulation in 49 genes, featuring 15 genes with increased expression and 34 genes with decreased expression. In parallel, Formulation 3 demonstrated regulatory impacts on 62 genes, with 13 genes experiencing upregulation and 49 genes undergoing downregulation.

In further analyzing the data obtained for the upregulated genes, the formulations tested demonstrate a consistent elevation in the activity of DNAJB11, ICAM1, KRT8, SCD, SERPINE1, TMEM2, and TXNRD1. Especially, the DNAJB11 gene encodes a member of the DNAJ/HSP40 protein family, serving as a co-chaperone pivotal in protein folding and quality control within the endoplasmic reticulum (ER) function. Its involvement in regulating the unfolded protein response (UPR) and ER-associated degradation (ERAD) highlights its significance in maintaining cellular proteostasis, impacting cellular metabolic pathways directly (28, 32). While natural products with antioxidant and anti-inflammatory properties are extensively studied for their potential to modulate ER stress responses and associated chaperone proteins, specific data on natural products directly upregulating the DNAJB11 gene remains elusive.

ICAM1, encoding a cell surface glycoprotein is essential in immune responses and inflammation, facilitating the adhesion of immune cells to endothelial cells. It plays a crucial role in immune responses by aiding in leukocyte endothelial transmigration during inflammation. Numerous natural products, such as resveratrol (33) or *Sphaeranthus indicus* (31), have been previously scrutinized for their positive impacts on immune responses. Conversely, KRT8, a type II keratin family member, maintains the structural integrity of epithelial cells and regulates cell growth and apoptosis signaling (34). While natural products with antioxidant and anti-inflammatory properties are commonly investigated for their potential to modulate cell apoptosis, information about natural products directly upregulating the KRT8 gene remains scarce. TMEM2, a transmembrane protein with diverse cellular functions, is associated in various cellular mechanisms (35, 36). To date, specific information about natural products directly upregulating the TMEM2 gene remains absent. Regarding the SCD, it participates in the biosynthesis of monounsaturated fatty acids, holding a pivotal role in lipid metabolism. Its associations with pathways related to fatty acid metabolism, lipid biosynthesis, and energy homeostasis are well-documented (16). Over the last decade, however, certain oil-based products have been examined for their favorable role in lipid biosynthesis (37, 38).

SELENBP1, involved in selenium transport and metabolism, is associated with pathways related to selenium metabolism, redox regulation, and antioxidant defense (39, 40). While specific natural products upregulating this gene are limited, sporadic research has explored the impact of selenium-rich diets (41). Finally, the TXNRD1, an enzyme integral to redox homeostasis within the thioredoxin system, plays a crucial role in pathways related to cellular redox balance, antioxidant defense, and the regulation of various cellular processes (42, 43). Limited studies have explored the influence of natural products on the expression of the TXNRD1 gene, with few exceptions such as garlic (44) and certain antioxidants (32).

Certain genes, including ASAH1, FASN, HYOU1, and ACTB, exhibit exclusive upregulation in response to Formulations, indicating a nuanced regulatory response to the different compositions. ASAH1, encoding acid ceramidase, plays a crucial role in sphingolipid metabolism by hydrolyzing ceramide into sphingosine and fatty acid, thereby influencing cellular processes such as apoptosis, cell growth, and inflammation (45–47). HYOU1, functions as a molecular chaperone under hypoxic and endoplasmic reticulum (ER) stress conditions, modulates protein folding and cell survival, although limited literature exists on natural products influencing HYOU1 upregulation (48–50). Likewise, GCLC, encoding the catalytic subunit of glutamate-cysteine ligase, plays a crucial role in cellular redox balance and antioxidant defense, with limited studies exploring the impact of natural products on GCLC expression, notably resveratrol (51).

ACTB, as a cytoskeletal protein, participates in maintaining cell structure and facilitating cell movement highlighting its involvement in various cellular processes, including cell motility and division (51, 52). Although extensive reviews have been conducted, on the cytoskeletal effects of natural products research specifically targeting ACTB expression is limited (34). Lastly with LGR5, acting as a receptor it is involved in Wnt signaling and a marker for adult stem cells, holding promise for tissue regeneration and stem cell population maintenance. However, there is currently a lack of literature analyzing the impact of natural products on LGR5 expression, suggesting a potential area for future investigation (53, 54).

The findings obtained in this study highlight the potential role of olive oil fatty acid composition as a ligand for the PPAR alpha pathway, offering insights into its therapeutic implications. Specifically, the observed upregulation of genes associated with lipid metabolism, such as FASN (fatty acid synthase) and SCD (stearoyl-CoA desaturase), underscores the influence of olive oil fatty acids on critical enzymes involved in the *de novo* lipogenesis and the synthesis of monounsaturated fatty acids. FASN plays a central role in catalyzing the synthesis of long-chain fatty acids, contributing to lipid biosynthesis and energy storage processes (55–57). Its upregulation suggests an enhancement of *de novo* lipogenesis, potentially leading to altered lipid metabolism and energy homeostasis. On the other hand, SCD catalyzes the conversion of saturated fatty acids into monounsaturated fatty acids, playing a key role in regulating the saturation status of cellular lipids (58). Upregulation of SCD indicates an increase in the synthesis of monounsaturated fatty acids, which are known to possess anti-inflammatory and cardioprotective properties. These fatty acids serve as endogenous ligands for the PPAR alpha pathway, a nuclear receptor involved in the transcriptional regulation of genes related to lipid metabolism, inflammation, and oxidative stress. Therefore, the modulation of FASN and SCD expression by olive oil fatty acids suggests a potential mechanism by which they exert their beneficial effects on lipid metabolism, inflammation, and oxidative stress. Further investigation into the specific fatty acid composition of olive oil and its interactions with the PPAR alpha pathway may offer valuable insights into the development of novel therapeutic strategies targeting metabolic disorders and inflammatory conditions.

Importantly, distinct formulations elicit independent responses from specific genes, highlighting the nuanced regulatory effects of each formulation. Formulation 1 uniquely influences CDKN1A, HMOX1, NQO1, and PSME3. CDKN1A, known for its role in cell cycle regulation and tumor suppression, governs cell proliferation dynamics (59). HMOX1 contributes to heme degradation, participating in heme metabolism and cellular antioxidant defense mechanisms (60). Additionally, NQO1 plays a crucial role in detoxification by reducing quinones, preventing cellular oxidative damage (61, 62). PSME3, a constituent of the 26S proteasome, is essential for protein degradation and cellular homeostasis (63, 64). While existing research primarily focuses on the anticancer activities of natural products targeting these genes (65), their potential antioxidative roles in brain diseases (66), and promising phytobioactives (67), warrant further exploration.

Concurrently, Formulation 2 selectively impacts genes CCNG1, PYGL, and SERPINA3. CCNG1, involved in cell cycle progression, regulates cell cycle arrest and apoptosis (68). PYGL, participates in glycogen metabolism-gluconeogenesis, catalyzing the breakdown of glycogen into glucose-1-phosphate (69, 70). SERPINA3 modulates inflammatory responses through protease inhibition (71). Although these genes play significant roles in various cellular pathways, comprehensive research on the impact of natural products on their expression is limited, necessitating further investigation (15). Regarding Formulation 3-induced upregulation genes like ABCB1, ABCC2 ALDOA, and SLC2A3 are involved in drug metabolism, cellular detoxification, glycolysis, and glucose transportation. ABCB1 and ABCC2 encode membrane transporters pivotal for controlling intracellular levels of various xenobiotics and drugs (72, 73). ALDOA and SLC2A3 contribute to glycolysis and glucose uptake, respectively (74–76). While existing studies highlight the impact of natural products on these genes, particularly in anticancer effects, multidrug resistance modulation, and glucose transportation, further investigations are imperative for a more comprehensive understanding of their therapeutic implications (18, 77–80).

In the context of downregulated genes, a noteworthy decrease was observed in the expression levels of AVPR1A, COL4A1, CXCL12, FMO1, PDYN, S100A8, HAO2, and THRSP genes. Specifically, AVPR1A, responsible for encoding a vasopressin receptor implicated in water reabsorption and vasoconstriction, exhibited a substantial reduction in expression (81). Similarly, COL4A1, a gene encoding a crucial component of type IV collagen essential for basement membrane structural integrity, demonstrated a significant decrease in expression (82). CXCL12, contributing to immune cell migration and hematopoiesis through its role as a chemokine, and PDYN, encoding a precursor protein for dynorphins, also displayed noteworthy reductions in expression (83, 84). FMO1, an enzyme pivotal in xenobiotic metabolism, showcased diminished expression levels (85, 86). Furthermore, S100A8, functioning in the regulation of inflammatory processes as a calcium-binding protein, and HAO2 encoding a protein mainly involved in the oxidation of medium and long chain hydroxyacids, both exhibited notable reductions in expression (87, 88). Lastly, THRSP, a gene regulating thyroid hormone responsiveness, displayed a significant decrease in expression (89).

Concerning the correlation of these genes with natural products, limited experimental investigations have been identified, primarily focusing on anticancer activities, neuroprotection, inflammatory response modulation, and thyroid hormone regulation influenced by natural products (90–94). While the current body of research provides valuable insights into the potential impact of natural products on these downregulated genes, especially in the context related to cancer, neuroprotection, inflammatory responses, and thyroid hormone regulation, further exploration is warranted to unravel the intricate molecular mechanisms underlying these associations.

The gene ontology analysis reveals distinct effects of each formulation on biological processes, molecular functions, and cellular components. Formulation 1 predominantly shapes lipid metabolism and growth factor binding, while Formulation 2 influences transmembrane activities and cellular matrix interactions, correlating with conditions like type 2 diabetes and metabolic disorders. Formulation 3 notable affects fatty acid and lipid metabolism, alongside transportation processes, highlighting the need for tailored therapeutic approaches. Furthermore, the intricate network of gene regulation suggests the formulations’ potential to counteract oxidative stress, efficiently metabolize antioxidant constituents, and influence fatty acid metabolism and cholesterol regulation.

In the context of AD pathophysiology, the alterations in the expression of ASAH1, CDKN1A, and DNAJB11 are notable, since these genes have gained attention due to their roles in cellular stress responses and protein quality control mechanisms. ASAH1 encodes acid ceramidase involved in ceramide metabolism, which has been implicated in neuronal apoptosis and neuroinflammation, both hallmarks of AD pathology (40, 41). Similarly, CDKN1A, known as p21, is involved in cell cycle regulation and has been linked to neuronal apoptosis and synaptic dysfunction in AD brains (3). DNAJB11, a member of the HSP40 family of chaperones, participates in protein folding and degradation pathways, thereby influencing the aggregation and clearance of misfolded proteins implicated in AD, such as amyloid-beta and tau (95).

Genes associated with lipid metabolism, including FASN and SCD, contribute to AD pathogenesis by affecting amyloid-beta production and clearance pathways, contributing to neuroinflammation and synaptic dysfunction (59, 96). Additionally, antioxidant enzymes such as GCLC, HMOX1, and TXNRD1 is shown to play a crucial role in protecting neurons from oxidative stress, a prominent feature of AD pathology. Glutamate-cysteine ligase catalytic subunit (GCLC) and heme oxygenase-1 (HMOX1) are involved in the cellular response to oxidative stress and inflammation, while thioredoxin reductase 1 (TXNRD1) plays a crucial role in maintaining redox homeostasis (43, 97). Furthermore, genes involved in cellular adhesion and inflammation, such as ICAM1, can promote neuroinflammation and disrupt blood–brain barrier integrity, thereby exacerbating AD pathological maifestation (28). Additionally, ABC transporters, including ABCB1 and ABCC2, are involved in the efflux of amyloid-beta peptides from the brain, and their dysregulation may contribute to amyloid-beta accumulation and neurotoxicity in AD (78). Finally, aldolase A (ALDOA), and leucine-rich repeat-containing G-protein coupled receptor 5 (LGR5) are implicated in neuronal energy metabolism, synaptic plasticity, and neurogenesis, suggesting their involvement in AD pathophysiology (98). Understanding the complex interactions between these genes may pave the way for novel therapeutic strategies for AD.

While this study presents promising data on minimal hepatotoxicity and potential health benefits of olive-oil-based formulations, a comprehensive discussion on the limitations of the current research and suggestions for future studies is warranted. The *in vitro* nature of the study poses a significant limitation, as cellular responses observed in the controlled laboratory settings do not fully reflect the complex interactions that occur in living organisms. Therefore, validation through *in vivo* studies is essential to confirm the findings and assess the effects of the Formulations within the physiological context. Additionally, the inherent variability in natural product composition poses a challenge, as the bioactive components of olive oil and natural constituents may vary depending on factors such as geographical location, harvesting methods, and processing techniques. Applying gene array analysis to assess molecular aspects of hepatotoxicity of multi-nutrient formulation mixtures provides a systematic approach to unraveling the intricate interactions between complex botanical compounds and cellular pathways, essential in understanding their safety and efficacy. However, addressing these variations and understanding their impact is crucial for translating these findings into health and clinical applications effectively.

Moreover, further investigation is needed to elucidate the specific pathways and molecular targets seen in the multi-nutrient formulations tested in this work. Studying the effects of olive-oil-based formulations over time and testing them in various cell models representing different tissues and disease conditions would provide valuable insights into their broad spectrum of therapeutic applications. Furthermore, investigating the mechanisms by which the antioxidant compounds in olive oil exert their effects would aid in optimizing formulation strategies for enhanced efficacy. In conclusion, while the current study sheds light on the promising potential of olive-oil-based formulations, acknowledging the limitations and opportunities for future research is essential to pave the way for the development of safer, more effective natural-based nutraceuticals and multi-nutrient health supplements.

## Data availability statement

The original contributions presented in the study are included in the article/Supplementary material, further inquiries can be directed to the corresponding author.

## Ethics statement

Ethical approval was not required for the studies on humans in accordance with the local legislation and institutional requirements because only commercially available established cell lines were used.

## Author contributions

SP: Conceptualization, Data curation, Investigation, Methodology, Writing – original draft, Writing – review & editing. FC: Writing – review & editing, Resources, Methodology, Data curation. AS: Data curation, Methodology, Writing – original draft, Writing – review & editing. AG-D: Writing – review & editing, Writing – original draft, Methodology, Data curation. LK: Investigation, Methodology, Writing – review & editing. AP: Writing – review & editing. DC: Writing – review & editing. VV: Writing – review & editing. IV: Conceptualization, Data curation, Funding acquisition, Investigation, Project administration, Resources, Supervision, Writing – original draft, Writing – review & editing.
