# Supplementary material for: Hepatotoxicity assessment of innovative nutritional supplements based on olive-oil formulations enriched with natural antioxidants
Source: Front Nutr. 2024 May 15;11:1388492. doi: 10.3389/fnut.2024.1388492 (PMC11133736; doi:10.3389/fnut.2024.1388492)
Supplement: Supplementary file 1 [file Table_1.pdf]

Supplementary Material

Hepatotoxicity Assessment of Innovative Nutritional Supplements Based on Olive-Oil Formulations Enriched with Natural Antioxidants

Sofia I. Prodromou[1], Fani Chatzopoulou[2,3], Aikaterini Saiti[1], Alexandros Giannopoulos-Dimitriou[1], Loukia A. Koudoura[1], Anastasia A. Pantazaki[4], Dimitrios Chatzidimitriou[2], Vasilis Vasiliou[5], Ioannis S. Vizirianakis[1,6*]

[1]Laboratory of Pharmacology, School of Pharmacy, Aristotle University of Thessaloniki, 54124 Thessaloniki, Greece

[2]Laboratory of Microbiology, School of Medicine, Aristotle University of Thessaloniki, 54124 Thessaloniki, Greece

[3]Labnet Laboratories, Department of Molecular Biology and Genetics, 54638 Thessaloniki, Greece

[4]Laboratory of Biochemistry, Department of Chemistry, Aristotle University of Thessaloniki, 54124 Thessaloniki, Makedonia, Greece

[5]Department of Environmental Health Sciences, Yale School of Public Health, Yale University, New Haven, CT 06520, USA

[6]Department of Health Sciences, School of Health and Life Sciences, University of Nicosia, Nicosia CY-1700, Cyprus

*** Correspondence:**
Ioannis S. Vizirianakis
<ivizir@pharm.auth.gr> or <vizirianakis.i@unic.ac.cy>

1 Supplementary Tables

Supplementary Table 1. Up-regulated genes and pathways affected in HepG2 cells exposed to formulation 1 (the KEGG database was used for pathway analysis).

Formulation 1							
**Gene_ID**	**Pathways**						
**ASAH1**	Sphingolipid metabolism	Lysosome	Sphingolipid signaling pathway				
**CDKN1A**	Transcriptional misregulation in cancer	Cell cycle	Viral carcinogenesis	Parathyroid hormone synthesis, secretion and action			
**DNAJB11**	Protein processing in endoplasmic reticulum						
**FASN**	Fatty acid metabolism / biosynthesis	Metabolic pathways	AMPK signaling pathway	Insulin signaling pathway	Alcoholic liver disease		
**GCLC**	Biosynthesis of cofactors	Metabolic pathways	Cysteine and methionine metabolism	Glutathione metabolism	Longevity regulating pathway - worm	Ferroptosis	
**HMOX1**	MicroRNAs in cancer	Metabolic pathways	Biosynthesis of secondary metabolites	Mineral absorption	Pathways in cancer	Chemical carcinogenesis - reactive oxygen species	Fluid shear stress and atherosclerosis
**HYOU1**	Protein processing in endoplasmic reticulum						
**ICAM1**	Cell adhesion molecules	Natural killer cell mediated cytotoxicity	TNF signaling pathway	Leukocyte transendothelial migration	Rheumatoid arthritis	AGE-RAGE signaling pathway in diabetic complications	Viral myocarditis
**KRT8**	Nervous system development	Keratinization					
**NQO1**	Biosynthesis of cofactors	Chemical carcinogenesis - reactive oxygen species	Ubiquinone and other terpenoid-quinone biosynthesis	Biosynthesis of secondary metabolites	Pathways in cancer		

**PSME3**	Proteasome	Antigen processing and presentation	Hepatitis C				
**SCD**	Biosynthesis of unsaturated fatty acids	Fatty acid metabolism	Biosynthesis of nucleotide sugars	PPAR signaling pathway	Amino sugar and nucleotide sugar metabolism	Naphthalene degradation	
**SERPINE1**	HIF-1 signaling pathway	Cellular senescence	Apelin signaling pathway	Complement and coagulation cascades	AGE-RAGE signaling pathway in diabetic complications		
**TMEM2**	Various types of N-glycan biosynthesis	MAPK signaling pathway - plant	Protein processing in endoplasmic reticulum				
**TXNRD1**	RNA Polymerase II Transcription	Nuclear events mediated by NFE2L2	Bacterial Infection Pathways	Selenoamino acid metabolism			
**ACTB**	ATP-dependent chromatin remodeling	Leukocyte transendothelial migration	Bacterial invasion of epithelial cells	Proteoglycans in cancer	Motor proteins	Apoptosis	Oxytocin signaling pathway

Supplementary Table 2. Up-regulated genes and pathways affected in HepG2 cells exposed to formulation 2 (the KEGG database was used for pathway analysis).

Formulation 2							
**Gene_ID**	**Pathways**						
**ASAH1**	Sphingolipid metabolism	Lysosome	Sphingolipid signaling pathway				
**CCNG1**	MicroRNAs in cancer	p53 signaling pathway					
**DNAJB11**	Protein processing in endoplasmic reticulum						
**FASN**	Fatty acid metabolism / biosynthesis	Metabolic pathways	AMPK signaling pathway	Insulin signaling pathway	Alcoholic liver disease		
**HYOU1**	Protein processing in endoplasmic reticulum						
**ICAM1**	Cell adhesion molecules	Natural killer cell mediated cytotoxicity	TNF signaling pathway	Leukocyte transendothelial migration	Rheumatoid arthritis	AGE-RAGE signaling pathway in diabetic complications	Viral myocarditis
**KRT8**	Nervous system development	Keratinization					
**LGR5**	Wnt signaling pathway						
**PYGL**	Necroptosis	Glucagon signaling pathway					
**SCD**	Biosynthesis of unsaturated fatty acids	Fatty acid metabolism	Biosynthesis of nucleotide sugars	PPAR signaling pathway	Amino sugar and nucleotide sugar metabolism	Naphthalene degradation	
**SERPINA3**	Innate Immune System	Interleukin-6 family signaling	Response to elevated platelet cytosolic Ca2+				
**SERPINE1**	HIF-1 signaling pathway	Cellular senescence	Apelin signaling pathway	Complement and coagulation cascades	AGE-RAGE signaling pathway in diabetic complications		

**TMEM2**	Various types of N-glycan biosynthesis	MAPK signaling pathway - plant	Protein processing in endoplasmic reticulum				
**TXNRD1**	RNA Polymerase II Transcription	Nuclear events mediated by NFE2L2	Bacterial Infection Pathways	Selenoamino acid metabolism			
**ACTB**	ATP-dependent chromatin remodeling	Leukocyte transendothelial migration	Bacterial invasion of epithelial cells	Proteoglycans in cancer	Motor proteins	Apoptosis	Oxytocin signaling pathway

Supplementary Table 3. Up-regulated genes and pathways affected in HepG2 cells exposed to formulation 3 (the KEGG database was used for pathway analysis).

**Formulation 3**							
**Gene_ID**	**Pathways**						
**ABCB1**	ABC transporters	Cholesterol metabolism	Endocrine resistance	MicroRNAs in cancer			
**ABCC2**	Antifolate resistance	ABC transporters	Platinum drug resistance				
**ALDOA**	HIF-1 signaling pathway						
**DNAJB11**	Protein processing in endoplasmic reticulum						
**GCLC**	Biosynthesis of cofactors	Metabolic pathways	Cysteine and methionine metabolism	Glutathione metabolism	Longevity regulating pathway - worm	Ferroptosis	
**ICAM1**	Cell adhesion molecules	Natural killer cell mediated cytotoxicity	TNF signaling pathway	Leukocyte transendothelial migration	Rheumatoid arthritis	AGE-RAGE signaling pathway in diabetic complications	Viral myocarditis
**KRT8**	Nervous system development	Keratinization					
**LGR5**	Wnt signaling pathway						
**SCD**	Biosynthesis of unsaturated fatty acids	Fatty acid metabolism	Biosynthesis of nucleotide sugars	PPAR signaling pathway	Amino sugar and nucleotide sugar metabolism	Naphthalene degradation	
**SERPINE1**	HIF-1 signaling pathway	Cellular senescence	Apelin signaling pathway	Complement and coagulation cascades	AGE-RAGE signaling pathway in diabetic complications		
**SLC2A3**	**Gene expression (Transcription)**	**Innate Immune System**	Metabolism of water-soluble vitamins and cofactors	Transport of inorganic cations/anions and amino acids/oligopeptides	Nuclear receptors meta-pathway		

**TMEM2**	Various types of N-glycan biosynthesis	MAPK signaling pathway - plant	Protein processing in endoplasmic reticulum				
**TXNRD1**	RNA Polymerase II Transcription	Nuclear events mediated by NFE2L2	Bacterial Infection Pathways	Selenoamino acid metabolism			

Supplementary Table 4. Gene expression alterations in HepG2 cells exposed to different treatments of formulations 1, 2 and 3, as detected by qPCR analysis using the RT2 Profiler PCR Array.

Symbol	Description	Fold Change	Fold Up- or Down-Regulation	Fold Change	Fold Up- or Down-Regulation	Fold Change	Fold Up- or Down-Regulation	Action of protein	Related Pathways
		Formulation 1 /Control Group	Formulation 1	Formulation 2 /Control Group	Formulation 2	Formulation 3 /Control Group	Formulation 3		
ABCB1	ATP-binding cassette, sub-family B (MDR/TAP), member 1	1,70	1,70	**3,71**	**3,71**	1,34	1,34	Multidrug resistance.	Imipramine/Desipramine Pathway, Pharmacokinetics and Drug ADME.
ABCB11	ATP-binding cassette, sub-family B (MDR/TAP), member 11	**0,00**	**-219,87**	**0,00**	**-1786,72**	**0,00**	**-894,36**	Multidrug resistance (major canalicular bile salt export pump)	Synthesis of bile acids and bile salts and Statin Pathway-Generalized, Pharmacokinetics.
ABCB4	ATP-binding cassette, sub-family B (MDR/TAP), member 4	**0,05**	**-20,77**	**0,06**	**-17,91**	**0,18**	**-5,45**	Transport of phospholipids from liver hepatocytes into bile.	Regulation of activated PAK-2p34 by proteasome mediated degradation and PPARA activates gene expression.
ABCC2	ATP-binding cassette, sub-family C (CFTR/MRP), member 2	1,49	1,49	**2,32**	**2,32**	1,58	1,58	Canalicular Multidrug Resistance Protein	Drug ADME and Statin Pathway-Generalized, Pharmacokinetics.
ABCC3	ATP-binding cassette, sub-family C (CFTR/MRP), member 3	0,86	-1,16	**0,42**	**-2,40**	0,60	-1,66	Canicular Multispecific Organic Anion Transporter	Drug ADME and Paracetamol ADME.
ALDOA	Aldolase A, fructose-bisphosphate	1,36	1,36	**4,31**	**4,31**	1,27	1,27	Epididymis Secretory Sperm Binding Protein Li 61p	glycolysis (BioCyc) and Response to elevated platelet cytosolic Ca2+
APEX1	APEX nuclease (multifunctional DNA repair enzyme) 1	0,64	-1,57	**0,35**	**-2,85**	0,86	-1,16	Multifunctional DNA Repair Enzyme	Base excision repair and Packaging Of Telomere Ends
ASAH1	N-acylsphingosine amidohydrolase (acid ceramidase) 1	**2,45**	**2,45**	1,75	1,75	**3,03**	**3,03**	Acid Ceramidase	Innate Immune System and Sphingolipid metabolism
ATP8B1	ATPase, aminophospholipid transporter, class I, type 8B, member 1	**0,13**	**-8,00**	**0,07**	**-14,95**	**0,18**	**-5,62**	Probable Phospholipid-Transporting ATPase	Ion channel transport and Synthesis of bile acids and bile salts.

AVPR1A	Arginine vasopressin receptor 1A	**0,00**	**-1112,80**	**0,00**	**-1334,85**	**0,00**	**-894,36**	Antidiuretic Hormone Receptor 1A	Class A/1 (Rhodopsin-like receptors) and GPCR downstream signalling.
BHMT	Betaine--homocysteine S-methyltransferase	**0,02**	**-41,54**	**0,03**	**-38,94**	**0,10**	**-10,25**	Epididymis Secretory Sperm Binding Protein Li 61p	superpathway of choline degradation to L-serine and One-carbon metabolism and related pathways.
BTG2	BTG family, member 2	**0,04**	**-25,69**	**0,01**	**-79,31**	**0,05**	**-19,26**	Nerve Growth Factor-Inducible Anti-Proliferative	Gene expression (Transcription) and TP53 Regulates Transcription of Cell Cycle Genes.
CA3	Carbonic anhydrase III, muscle specific	**0,01**	**-68,23**	**0,01**	**-89,60**	**0,02**	**-58,87**	Epididymis Secretory Sperm Binding Protein Li 167mP (tissue specific: high levels in skeletal muscle and lower levels in cardiac and smooth muscle.)	Metabolism and Reversible hydration of carbon dioxide.
CASP3	Caspase 3, apoptosis-related cysteine peptidase	1,19	1,19	**0,48**	**-2,09**	0,95	-1,05	Apoptosis-Related Cysteine Peptidase	TNFR1 Pathway and Apoptosis and survival FAS signaling cascades.
CCNG1	Cyclin G1	1,69	1,69	1,43	1,43	**2,46**	**2,46**	cyclin-dependent protein kinases	Gene expression (Transcription) and Regulation of TP53 Expression and Degradation.
CD36	CD36 molecule (thrombospondin receptor)	**0,03**	**-36,67**	**0,05**	**-21,35**	**0,08**	**-12,15**	fourth major glycoprotein of the platelet surface-receptor for thrombospondin	MyD88 dependent cascade initiated on endosome and Diseases of Immune System.
CD68	CD68 molecule	**0,30**	**-3,32**	**0,17**	**-5,85**	**0,08**	**-12,63**	Scavenger Receptor-monocytes and tissue macrophages	Innate Immune System and Macrophage markers.
CDC14B	CDC14 cell division cycle 14 homolog B (S. cerevisiae)	**0,33**	**-3,02**	**0,40**	**-2,50**	0,51	-1,98	Dual Specificity Protein Phosphatase-exit of cell mitosis and initiation of DNA replication	RAF/MAP kinase cascade and Regulation of activated PAK-2p34 by proteasome mediated degradation.
CDKN1 A	Cyclin-dependent kinase inhibitor 1A (p21, Cip1)	**3,02**	**3,02**	1,98	1,98	0,73	-1,36	CDK-Interacting Protein 1	Regulation of activated PAK-2p34 by proteasome mediated degradation and Aberrant regulation of mitotic G1/S transition in cancer due to RB1 defects.
COL4A1	Collagen, type IV, alpha 1	**0,00**	**-1112,80**	**0,00**	**-1786,72**	**0,00**	**-894,36**	Collagen Alpha-1(IV) Chain protein-integral components of basement membranes	Collagen chain trimerization and Integrin Pathway.

CRYL1	Crystallin, lambda 1	**0,43**	**-2,30**	**0,13**	**-7,51**	1,17	1,17	Testicular Tissue Protein Li 44-alternative glucose metabolic pathway	Formation of xylulose-5-phosphate and Glycosaminoglycan metabolism.
CXCL12	Chemokine (C-X-C motif) ligand 12	**0,00**	**-1112,80**	**0,00**	**-1786,72**	**0,00**	**-894,36**	Stromal Cell-Derived Factor 1	Apoptotic Pathways in Synovial Fibroblasts and MIF Mediated Glucocorticoid Regulation.
CYP1A2	Cytochrome P450, family 1, subfamily A, polypeptide 2	**0,01**	**-185,04**	**0,00**	**-268,12**	**0,00**	**-249,00**	Cytochrome P450 4 superfamily of enzymes	Imipramine/Desipramine Pathway, Pharmacokinetics and Metapathway biotransformation Phase I and II.
DDIT4L	DNA-damage-inducible transcript 4-like	**0,00**	**-1112,80**	**0,00**	**-275,65**	**0,00**	**-894,36**	DNA-Damage-Inducible Transcript 4	MTOR signalling.
DDX39A	DEAD (Asp-Glu-Ala-Asp) box polypeptide 39A	0,98	-1,02	1,46	1,46	0,70	-1,43	Nuclear RNA Helicase	Gene expression (Transcription) and Transport of Mature Transcript to Cytoplasm.
DNAJB11	DnaJ (Hsp40) homolog, subfamily B, member 11	**2,42**	**2,42**	**2,29**	**2,29**	**2,28**	**2,28**	Human DnaJ Protein 9- co-chaperone of binding immunoglobulin protein	Unfolded Protein Response (UPR) and Cellular responses to stimuli.
DNAJC3	DnaJ (Hsp40) homolog, subfamily C, member 3	0,51	-1,96	0,67	-1,48	0,59	-1,69	Double-Stranded RNA-Activated Protein Kinase Inhibitor	Unfolded Protein Response (UPR) and Innate Immune System.
FABP1	Fatty acid binding protein 1, liver	**0,08**	**-13,16**	**0,05**	**-19,16**	0,84	-1,19	Liver-Type Fatty Acid-Binding Protein	Cellular responses to stimuli and Triglyceride metabolism.
FADS1	Fatty acid desaturase 1	0,55	-1,82	**0,31**	**-3,28**	1,65	1,65	Regulate unsaturation of fatty acids	docosahexaenoate biosynthesis IV (4-desaturase, mammals) and Fatty acid metabolism.
EMC9	Family with sequence similarity 158, member A	0,62	-1,63	0,62	-1,61	1,39	1,39	**ER Membrane Protein Complex Subunit 9**	Undetermined-Contributes to membrane insertase activity.
FASN	Fatty acid synthase	**3,89**	**3,89**	0,78	-1,29	**12,05**	**12,05**	Catalyze the synthesis of palmitate from acetyl-CoA and malonyl-CoA	Metabolism of steroids and Metabolism of water-soluble vitamins and cofactors.
FMO1	Flavin containing monooxygenase 1	**0,00**	**-1112,80**	**0,00**	**-1786,72**	**0,00**	**-894,36**	catalyzes the oxidation of soft nucleophilic heteroatom centers in drugs, pesticides, and xenobiotics.	Metapathway biotransformation Phase I and II and Busulfan Pathway, Pharmacodynamics.

TIMM10 B	Fracture callus 1 homolog (rat)	**0,37**	**-2,72**	**0,08**	**-12,69**	**0,40**	**-2,53**	Translocase Of Inner Mitochondrial Membrane 10 Homolog B	Peroxisomal lipid metabolism.
GADD45 A	Growth arrest and DNA-damage-inducible, alpha	1,23	1,23	1,96	1,96	0,63	-1,58	Responds to environmental stresses	Endometrial cancer and Gene expression (Transcription).
GCLC	Glutamate-cysteine ligase, catalytic subunit	**4,38**	**4,38**	**4,32**	**4,32**	1,42	1,42	Gamma-Glutamylcysteine Synthetase	Glutathione conjugation and Nuclear events mediated by NFE2L2.
GSR	Glutathione reductase	0,51	-1,96	**0,21**	**-4,72**	0,79	-1,26	Reduces oxidized glutathione disulfide (GSSG) to the sulfhydryl form GSH, (cellular antioxidant).	Nuclear events mediated by NFE2L2 and Glutathione conjugation.
HAO2	Hydroxyacid oxidase 2 (long chain)	**0,00**	**-1112,80**	**0,00**	**-1786,72**	**0,00**	**-402,27**	Long Chain Alpha-Hydroxy Acid Oxidase	Peroxisomal lipid metabolism and Metabolism.
HMOX1	Heme oxygenase (decycling) 1	**3,27**	**3,27**	1,18	1,18	1,20	1,20	Essential enzyme in heme catabolism	Nuclear events mediated by NFE2L2 and Inflammasomes.
HPN	Hepsin	**0,12**	**-8,39**	**0,33**	**-3,01**	1,11	1,11	Transmembrane Protease Serine 1	MET promotes cell motility and Signaling by MST1.
HYOU1	Hypoxia up-regulated 1	**3,68**	**3,68**	1,54	1,54	**4,13**	**4,13**	Oxygen Regulated Protein	Unfolded Protein Response (UPR) and Cellular responses to stimuli.
ICAM1	Intercellular adhesion molecule 1	**3,81**	**3,81**	**2,28**	**2,28**	**4,74**	**4,74**	Encodes a cell surface glycoprotein-Rhinovirus Receptor	Blood-Brain Barrier and Immune Cell Transmigration: VCAM-1/CD106 Signaling and Cytokine Signaling in Immune system.
IGFALS	Insulin-like growth factor binding protein, acid labile subunit	**0,00**	**-253,72**	**0,00**	**-364,83**	**0,02**	**-53,68**	Insulin-Like Growth Factor-Binding Protein Complex Acid Labile Subunit	Regulation of Insulin-like Growth Factor (IGF) transport and uptake by Insulin-like Growth Factor Binding Proteins (IGFBPs) and Metabolism of proteins.
IL6ST	Interleukin 6 signal transducer (gp130, oncostatin M receptor)	**0,33**	**-3,03**	**0,42**	**-2,36**	0,51	-1,96	Gp130 Of The Rheumatoid Arthritis Antigenic Peptide-Bearing Soluble Form	Interleukin-6 family signaling and IL27-mediated signaling events.
IPO4	Importin 4	1,23	1,23	**0,39**	**-2,53**	0,93	-1,08	Enable nuclear import signal receptor activity and nuclear localization sequence binding activity.	Undetermined

FAM214 A	KIAA1370	**0,30**	**-3,34**	**0,19**	**-5,20**	**0,17**	**-6,05**	Unknown- Probably immune role	Undetermined
KRT18	Keratin 18	1,19	1,19	1,61	1,61	1,02	1,02	Cytokeratin 18	Keratinization and Nervous system development.
KRT8	Keratin 8	**3,49**	**3,49**	**2,42**	**2,42**	**2,52**	**2,52**	Keratin, Type II Cytoskeletal 8	Keratinization and Nervous system development.
L2HGDH	L-2-hydroxyglutarate dehydrogenase	**0,24**	**-4,09**	**0,02**	**-46,17**	**0,19**	**-5,21**	Encodes L-2-hydroxyglutarate dehydrogenase	Pyruvate metabolism and Respiratory electron transport, ATP synthesis by chemiosmotic coupling, and heat production by uncoupling proteins.
LGR5	Leucine-rich repeat containing G protein-coupled receptor 5	1,46	1,46	**2,34**	**2,34**	**3,03**	**3,03**	G-Protein Coupled Receptor	Signaling by WNT and ncRNAs involved in Wnt signaling in hepatocellular carcinoma.
LPL	Lipoprotein lipase	**0,00**	**-1112,80**	**0,00**	**-1580,06**	**0,00**	**-894,36**	Encodes lipoprotein lipase	Plasma lipoprotein assembly, remodeling, and clearance and Familial hyperlipidemia type 1.
LSS	Lanosterol synthase (2,3-oxidosqualene-lanosterol cyclase)	**0,27**	**-3,67**	**0,09**	**-10,74**	1,96	1,96	Catalyzes the conversion of (S)-2,3 oxidosqualene to lanosterol	superpathway of cholesterol biosynthesis and Metabolism of steroids.
MAOB	Monoamine oxidase B	1,39	1,39	**0,29**	**-3,45**	0,69	-1,46	Adrenalin Oxidase- metabol ism of neuroactive and vasoactive amines	Oxidation by cytochrome P450 and ethanol degradation II.
MAP3K6	Mitogen-activated protein kinase kinase kinase 6	**0,14**	**-7,03**	**0,02**	**-44,25**	**0,08**	**-12,71**	Apoptosis Signal-Regulating Kinase 2	TCR Signaling (Qiagen) and Angiopoietin-like protein 8 regulatory pathway.
MBL2	Mannose-binding lectin (protein C) 2, soluble	**0,03**	**-30,03**	**0,03**	**-39,82**	**0,18**	**-5,50**	Important element in the innate immune system.	Complement cascade and SARS-CoV-2 Infection.
MCM10	Minichromosome maintenance complex component 10	**0,04**	**-22,62**	**0,04**	**-27,61**	**0,21**	**-4,81**	Involved in the initiation of eukaryotic genome replication.	Activation of the pre-replicative complex and Mitotic G1 phase and G1/S transition.
MLXIPL	MLX interacting protein-like	**0,27**	**-3,77**	**0,06**	**-15,69**	1,47	1,47	Williams Beuren Syndrome Chromosome Region 14	Integration of energy metabolism and Angiopoietin-like protein 8 regulatory pathway.
MRPS18 B	Mitochondrial ribosomal protein S18B	0,53	-1,87	**0,27**	**-3,77**	0,53	-1,88	Encoded by nuclear genes and help in protein synthesis within the mitochondrion.	Mitochondrial translation and Metabolism of proteins.

NQO1	NAD(P)H dehydrogenase, quinone 1	**2,03**	**2,03**	0,59	-1,69	1,57	1,57	Phylloquinone Reductase- encodes a cytoplasmic 2-electron reductase	Nuclear events mediated by NFE2L2 and Warfarin Pathway, Pharmacodynamics.
NUS1	Nuclear undecaprenyl pyrophosphate synthase 1 homolog (S. cerevisiae)	1,27	1,27	0,57	-1,75	1,77	1,77	Encodes a type I single transmembrane domain receptor for the neural and cardiovascular regulator Nogo-B.	Synthesis of substrates in N-glycan biosythesis and Diseases of glycosylation.
OSMR	Oncostatin M receptor	**0,47**	**-2,13**	1,01	1,01	**0,47**	**-2,15**	Encodes a member of the type I cytokine receptor family	Akt Signaling and Interleukin-6 family signaling.
SLC51A	Organic solute transporter alpha	**0,11**	**-9,52**	**0,17**	**-5,92**	**0,15**	**-6,61**	Enable protein heterodimerizatio n, homodimerization and transmembrane transporter activity	Synthesis of bile acids and bile salts and Metabolism
PDYN	Prodynorphin	**0,00**	**-1112,80**	**0,00**	**-1786,72**	**0,00**	**-894,36**	Proteolytically processed to form the secreted opioid peptides	GPCR downstream signalling and Class A/1 (Rhodopsin-like receptors).
PLA2G1 2A	Phospholipase A2, group XIIA	**0,28**	**-3,58**	**0,06**	**-16,01**	**0,25**	**-4,07**	Liberate arachidonic acid from phospholipids for production of eicosanoids and exert a variety of physiologic and pathologic effects.	Glycerophospholipid biosynthesis and Acyl chain remodelling of PE.
PPARA	Peroxisome proliferator-activated receptor alpha	**0,50**	**-2,00**	**0,31**	**-3,26**	1,06	1,06	Respiration-cholesterol and lipid metabolism.	Circadian Clock and Gene expression (Transcription).
PSME3	Proteasome (prosome, macropain) activator subunit 3 (PA28 gamma; Ki)	**2,07**	**2,07**	**0,43**	**-2,30**	1,53	1,53	Multicatalytic proteinase complex	Regulation of activated PAK-2p34 by proteasome mediated degradation and Assembly of the pre-replicative complex.
PYGL	Phosphorylase, glycogen, liver	0,76	-1,32	1,67	1,67	**2,16**	**2,16**	Glycogen Phosphorylase, Liver Form	Activation of cAMP-Dependent PKA and Innate Immune System.
RB1	Retinoblastoma 1	0,69	-1,44	1,31	1,31	1,03	1,03	Negative regulator of the cell cycle and was the first tumor suppressor gene found.	Aberrant regulation of mitotic G1/S transition in cancer due to RB1 defects and Regulation of activated PAK-2p34 by proteasome mediated degradation.
RDX	Radixin	1,53	1,53	1,32	1,32	1,09	1,09	Cytoskeletal protein that may be important in linking actin to	Sensory processing of sound and Nervous system development.

								the plasma membrane	
RHBG	Rh family, B glycoprotein (gene/pseudogen e)	**0,31**	**-3,26**	**0,05**	**-20,65**	1,08	1,08	Encodes one of two non-erythroid members of the Rhesus (Rh) protein family	Transport of inorganic cations/anions and amino acids/oligopeptides.
S100A8	S100 calcium binding protein A8	**0,00**	**-1112,80**	**0,00**	**-1786,72**	**0,00**	**-894,36**	Involved in cell cycle progression and differentiation.	MyD88 dependent cascade initiated on endosome and Diseases of Immune System.
SCD	Arylacetamide deacetylase	**3,95**	**3,95**	**18,91**	**18,91**	**18,59**	**18,59**	Involved in fatty acid biosynthesis, primarily the synthesis of oleic acid.	Metabolism of steroids and NR1H2 and NR1H3-mediated signaling.
SERPIN A3	Serpin peptidase inhibitor, clade A (alpha-1 antiproteinase, antitrypsin), member 3	0,59	-1,70	1,54	1,54	**6,86**	**6,86**	Inhibit serine proteases	Response to elevated platelet cytosolic Ca2+ and Innate Immune System.
SERPIN E1	Serpin peptidase inhibitor, clade E (nexin, plasminogen activator inhibitor type 1), member 1	**19,19**	**19,19**	**74,38**	**74,38**	**3,06**	**3,06**	Principal inhibitor of tissue plasminogen activator (tPA) and urokinase (uPA), and hence is an inhibitor of fibrinolysis.	Response to elevated platelet cytosolic Ca2+and Gene expression (Transcription).
SKIL	SKI-like oncogene	**0,10**	**-9,94**	**0,36**	**-2,76**	**0,09**	**-11,50**	Component of the SMAD pathway, which regulates cell growth	Gene expression (Transcription) and TGF-beta receptor signaling in skeletal dysplasias.
SLC17A 3	Solute carrier family 17 (sodium phosphate), member 3	**0,00**	**-519,21**	**0,00**	**-1786,72**	**0,00**	**-521,95**	Sodium-Dependent Phosphate Transport Protein 4	Ion channel transport and Transport of inorganic cations/anions and amino acids/oligopeptides.
SLC2A3	Solute carrier family 2 (facilitated glucose transporter), member 3	0,63	-1,58	**10,34**	**10,34**	**0,39**	**-2,57**	Enables dehydroascorbic acid, glucose binding and glucose transmembrane transporter activity.	Gene expression (Transcription) and Innate Immune System.
SLC39A 6	Solute carrier family 39 (zinc transporter), member 6	1,01	1,01	0,59	-1,71	0,82	-1,23	Show structural characteristics of zinc transporters	Metal ion SLC transporters and Transport of inorganic cations/anions and amino acids/oligopeptides.
SREBF1	Sterol regulatory element binding transcription factor 1	**0,13**	**-7,50**	**0,29**	**-3,48**	**0,36**	**-2,79**	Promoter of the LDL receptor gene-sterol biosynthesis.	Metabolism of steroids and Gene expression (Transcription).
TAGLN	Transgelin	**0,28**	**-3,61**	0,78	-1,29	1,30	1,30	Involved in calcium-independent	PDGFR-beta signaling pathway and Burn wound healing.

								smooth muscle contraction	
THRSP	Thyroid hormone responsive	**0,00**	**-1112,80**	**0,00**	**-1786,72**	**0,00**	**-894,36**	Lipogenic Protein	Metabolism and Fatty acid metabolism.
TMEM2	Transmembrane protein 2	**3,09**	**3,09**	**4,01**	**4,01**	**2,62**	**2,62**	Type II transmembrane protein	Undetermined
TXNRD1	Thioredoxin reductase 1	**15,03**	**15,03**	**15,01**	**15,01**	**4,01**	**4,01**	Encodes a member of the thioredoxin (Trx) system.	Nuclear events mediated by NFE2L2 and Gene expression (Transcription).
WIPI1	WD repeat domain, phosphoinositide interacting 1	1,06	1,06	0,53	-1,90	1,23	1,23	Encodes a WD40 repeat protein	Unfolded Protein Response (UPR) and Autophagy.
YRDC	YrdC domain containing (E. coli)	1,56	1,56	0,67	-1,49	0,92	-1,09	Enable nucleotidyltransferase activity and tRNA binding activity.	Undetermined
ACTB	Actin, beta	**2,36**	**2,36**	0,93	-1,08	**2,23**	**2,23**	Encodes one of six different actin proteins- involved in cell motility, structure, integrity, and intercellular signaling.	Signaling downstream of RAS mutants and Regulation of actin dynamics for phagocytic cup formation.
B2M	Beta-2-microglobulin	1,61	1,61	1,19	1,19	1,57	1,57	Encodes a serum protein found in MHC class I heavy chain on the surface of nearly all nucleated cells.	SARS-CoV-2 Infection and Antigen processing-Cross presentation.
GAPDH	Glyceraldehyde-3-phosphate dehydrogenase	**0,44**	**-2,28**	1,25	1,25	**0,47**	**-2,13**	Aging-Associated Gene 9 Protein	Glycolysis (BioCyc) and gluconeogenesis III.
HPRT1	Hypoxanthine phosphoribosyl transferase	1,21	1,21	1,19	1,19	1,04	1,04	central role in the generation of purine nucleotides through the purine salvage pathway.	Nucleotide salvage and Thiopurine Pathway, Pharmacokinetics/Pharmacodynamics.
RPLP0	Ribosomal protein, large, P0	**0,49**	**-2,03**	0,61	-1,63	0,58	-1,72	Neutral Ribosomal Phosphoprotein P0	Peptide chain elongation and rRNA processing in the nucleus and cytosol.

Note that: * To capture gene regulation in a way of multiple change (Fold Regulation) represents the effects of expression change in a biologically meaningful way. Change values (+) greater than one unit indicate positive or upward adjustment. Change values (-), less than one unit indicate negative or downward adjustment. Values greater than 2 units are indicated in red, and values less than 0.5, but also less than -2, are indicated in blue. In green color are indicated the genes that activated after the treatments with the formulations
